# Oleuropein confers neuroprotection against rotenone-induced model of Parkinson’s disease via BDNF/CREB/Akt pathway

**DOI:** 10.1038/s41598-023-29287-4

**Published:** 2023-02-11

**Authors:** Richa Singh, Walia Zahra, Saumitra Sen Singh, Hareram Birla, Aaina Singh Rathore, Priyanka Kumari Keshri, Hagera Dilnashin, Shekhar Singh, Surya Pratap Singh

**Affiliations:** Department of Biochemistry, Institute of Science, Banaras Hindu University, Varanasi, UP 221005 India

**Keywords:** Neuroscience, Diseases

## Abstract

Major pathological features of Parkinson’s disease (PD) include increase in oxidative stress leading to the aggregation of α-synuclein, mitochondrial dysfunction and apoptosis of dopaminergic neurons. In addition, downregulation of the expression of neurotrophic factors like-Brain Derived Neurotrophic Factor (BDNF) is also involved in PD progression. There has been a lot of interest in trophic factor-based neuroprotective medicines over the past few decades to treat PD symptoms. Rotenone, an insecticide, inhibits the mitochondrial complex I causing overproduction of ROS, oxidative stress, and aggregation of α-synuclein. It has been shown that BDNF and Tropomyosin receptor kinase B (TrkB) interaction initiates the regulation of neuronal cell development and differentiation by the serine/threonine protein kinases like Akt and GSK-3β. Additionally, Transcription factor CREB (cAMP Response Element-binding protein) also determines the gene expression of BDNF. The homeostasis of these signalling cascades is compromised with the progression of PD. Therefore, maintaining the equilibrium of these signalling cascades will delay the onset of PD. Oleuropein (OLE), a polyphenolic compound present in olive leaves has been documented to cross blood brain barrier and shows potent antioxidative property. In the present study, the dose of 8, 16 and 32 mg/kg body weight (bwt) OLE was taken for dose standardisation. The optimised doses of 16 and 32 mg/kg bwt was found to be neuroprotective in Rotenone induced PD mouse model. OLE improves motor impairment and upregulate CREB regulation along with phosphorylation of Akt and GSK-3β in PD mouse. In addition, OLE also reduces the mitochondrial dysfunction by activation of enzyme complexes and downregulates the proapoptotic markers in Rotenone intoxicated mouse model. Overall, our study suggests that OLE may be used as a therapeutic agent for treatment of PD by regulating BDNF/CREB/Akt signalling pathway.

## Introduction

Parkinson disease (PD) is the most prevalent neurodegenerative condition and movement disorder. The word parkinsonism refers to complex symptom that includes resting tremor, bradykinesia, and muscular stiffness^1,2^. The motor symptoms of PD are related to the degeneration of dopaminergic neurons (DA) in substantia nigra pars compacta (SNpc) and reduction of dopamine in striatum of mesencephalon. Although the existence of non-motor symptoms such as constipation, depression, sleep difficulties and cognitive impairment can also suggest neuronal loss in non-dopaminergic regions^3,4^. In persons 60 years and older, the prevalence of PD is estimated to be around 1%, rising from 1 to 3% in those aged 80 and above^5^. However, it is crucial to note that these figures do not include undiagnosed cases. The incidence of PD differs by gender, with a 3:2 ratio of males to females, with a delayed beginning in females attributable to neuroprotective action of estrogen on the nigrostriatal dopaminergic pathway^6^. Several cellular signalling pathways have been linked to the development of PD, with α-synuclein aggregation adopting a β-sheet-rich amyloid-like form being key to the progression of disease^7^. Previous studies have suggested that aberrant protein clearance, mitochondrial dysfunction, oxidative stress and neuroinflammation are all implicated in the genesis and progression of PD^8,9^. Brain-derived neurotrophic factor (BDNF), one of the neurotrophins (NTs) is involved in physiological function of the central nervous system (CNS). BDNF affects neuronal cell differentiation, survival, and neurite outgrowth of dopaminergic neurons, which is important for nervous system development^10,11^. It interacts with its major ligand TrkB through the immunoglobulin constant 2 (Ig-C2) domain, and this complex promotes neuronal survival by activating various downstream cascades via the phosphatidylinositol 3-kinase/Akt (PI3K/Akt) signalling pathways^12^. Akt is a major downstream kinase protein in BDNF/TrkB signalling which involved in neuronal cell survival^13^. When BDNF binds to the TrkB receptor, Akt is activated, while GSK-3 is deactivated due to phosphorylation at ser9, promoting cell survival. Activated Akt is also needed to phosphorylate CREB (cAMP Response Element-binding protein) at ser133 and convert it to its active form^14,15^. The BDNF gene contains CREB-binding sequences. CREB might be upstream of BDNF and upregulate BDNF expression. The survival, development, and synaptic plasticity of neurons are all affected by these cascades^16^.

Several investigations have shown that BDNF/TrkB signalling is involved in PD and have evaluated the possible therapeutic use of BDNF. TrkB is widely distributed in dopaminergic neurons of nigrostriatal region. Dopaminergic neuronal death is connected to pathogenic α-synuclein mutations that alter the interaction of BDNF with its receptor TrkB^17,18^. TrkB lipid raft distribution is suppressed by α-Synuclein, which also inhibits internalisation and axonal trafficking. α-Synuclein also inhibits TrkB production by binding with its kinase domain and causing its ubiquitination^19^. Therefore, reduced expression of BDNF and TrkB due to increase in oxidative stress and alpha synuclein aggregation was observed in PD^20^. Reduced p-Akt/Akt ratio is seen in the dopaminergic neurons of post-mortem PD brains, which is associated with PI3K/Akt signalling dysregulation and PD pathogenesis^21^. Degeneration of dopaminergic neurons by downregulation of BDNF, TrkB and Akt results in reducing the phosphorylation of CREB^22^. Thus, the activation of BDNF/CREB/Akt signalling may be studied to observe the neuroprotection against PD. Elevation of calcium (Ca^2+^) level also contribute to the oxidative stress-mediated PD leading to the $\alpha$-synuclein aggregation^23,24^. Neurotrophic BDNF may be helpful in decreasing the expression of a proapoptotic member of this family of proteins like Bax, Caspase3 and promote neuronal cell survival^25,26^.

Rotenone is a neurotoxin that inhibits mitochondrial electron transport chain (ETC) complex I and induces a parkinsonian like symptoms. It is being frequently used to study the behavioural features and molecular mechanisms of α-synuclein aggregation and degeneration of nigrostriatal dopaminergic neurons^27,28^. Currently number of drugs that are being used for the treatment of PD have several side effects. Therefore, novel treatment methods are required which can prevent the neuronal cell death and dysfunction. Therefore, natural plants and their structural analogue can serve as a principal source for the identification of novel drug for the treatment of PD^29^. Polyphenols in plants and fruits have been found to have antioxidant properties which prevents oxidative stress^30^. Polyphenols are abundant in olives and olive products^31^. The phenolic compound OLE is found in the olive leaf, oil, and fruit in large amounts^32^. OLE has almost 400% greater antioxidant capacity than vitamin C and double the antioxidant capacity of green tea or grape seed extract, and it may also pass the blood–brain barrier^33^. OLE has been shown to have anti-oxidative property in SN of the dopaminergic neurons in rats, as well as against 6-OHDA-induced PC12 cell damage^34^. In recent years many natural compounds have been shown to protect the degeneration of dopaminergic neurons via PI3K/Akt signalling pathway^35^. One possible challenge to find a significant dose for observing the neuroprotective effect of OLE through dose standardisation. In our experiment, the different concentration of OLE for dose standardisation was considered from Amira M. Badr et al. method^36^. OLE dosages of 16 mg/kg and 32 mg/kg bwt were both found to be significant. However, 16 mg/kg bwt was chosen for the subsequent research since it was the lowest dose demonstrating the best outcome.

As BDNF-CREB signalling pathway plays an important role in PD pathogenesis, therefore, this pathway can be targeted for therapeutic treatment of PD. OLE has been known to prevent the degeneration of neuron however, its interaction with different signalling pathways is still unknown. Hence, the present study was aimed to evaluate the neuroprotective effect of OLE on the neuronal growth and survival in rotenone induced parkinsonian mouse model via BDNF/CREB pathway.

## Dose chase experiment for the analysis of neuroprotective action of OLE

### Results 1

#### *OLE significantly mitigated motor impairment in Rotenone-induced parkinsonian mouse model*

**Figure 1:**
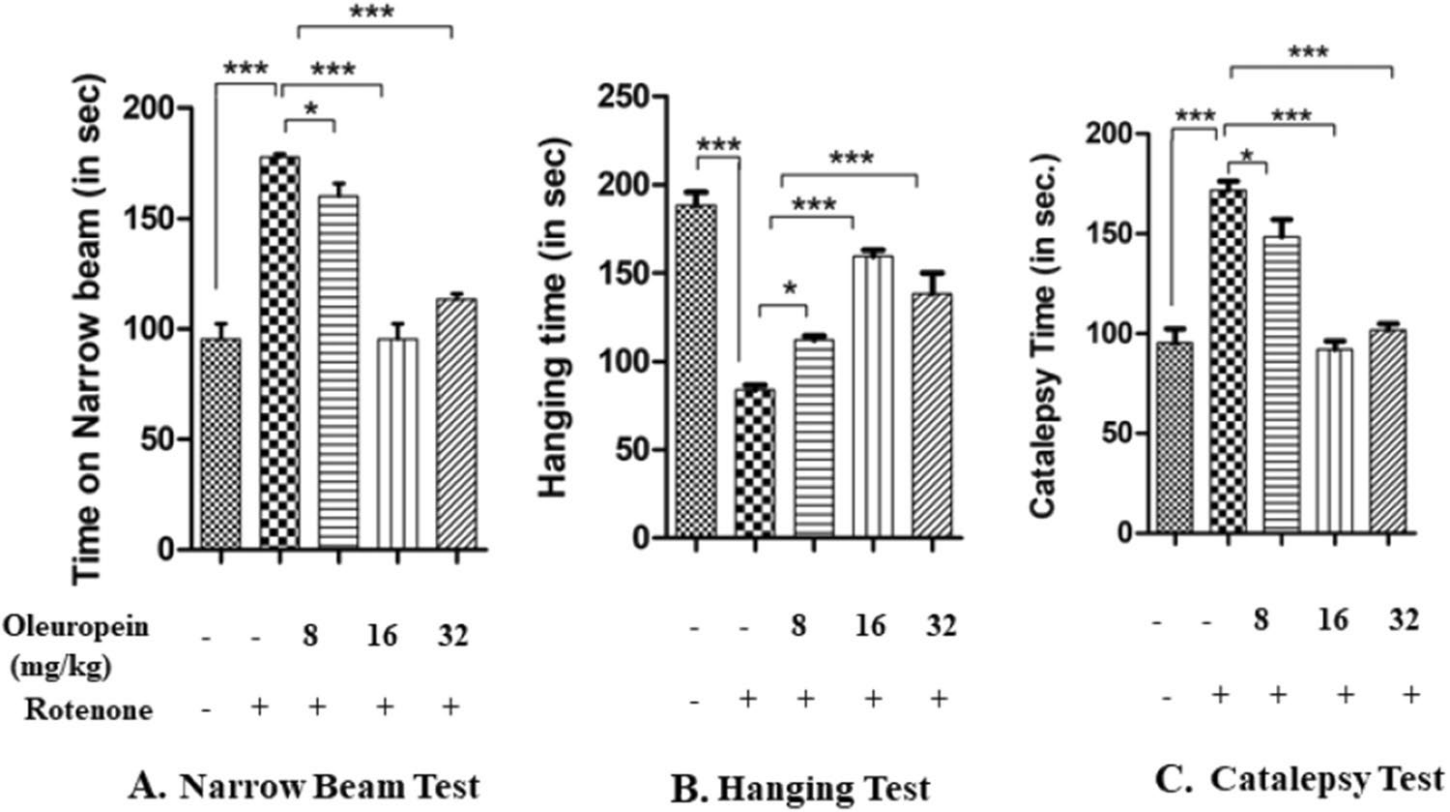
Effect of different doses of OLE on behavioural parameters in rotenone-induced mouse model. Narrow Beam Test showed increase in narrow beam walking time in rotenone group (***p < 0.001) as compared to the control group. Whereas, after the treatment with OLE (16 and 32 mg/kg bwt), the walking time of mice to cross the beam was significantly decreased (***p < 0.001). Hanging test presented decrease in hanging time in rotenone intoxicated mice as compared to control(***p < 0.001) whereas, in OLE treated group (16 and 32 mg/kg bwt), hanging time was significantly reduced (***p < 0.001). Catalepsy Test exhibited the reduced latency time to change its unusual posture was associated with rotenone induced mice (***p < 0.001) than control. While, significant decrease in catalepsy was observed in OLE treated mice (16 and 32 mg/kg bwt) (***p < 0.001).In all these three behavioural parameters, the OLE doses of 8 mg/kg bwt. showed a less significant effect. (*p < 0.05). The one-way ANOVA was used to analyse the data, followed by the Newman-Keuls test. The mean ± SEM (n = 6) is used to depict the values.

The observation of narrow beam walking test suggested that Rotenone intoxicated mice had a longer time to cross the beam (p < 0.001) as compared to control mice. While the treatment of OLE has significantly improved the behavioural abnormalities than Rotenone intoxicated PD mice model and took less time to cross the beam. Three doses of OLE (viz 8 mg/kg, 16 mg/kg and 32 mg/kg) were taken in the study. 16 mg/kg and 32 mg/kg bwt., doses of OLE were found to be significant (p < 0.001) (Fig. 1A). The Data are expressed in terms of mean ± SEM.

In the case of hanging test, the muscles strength and gripping power in Rotenone induced mice was significantly (p < 0.001) less than control group. Whereas in Rotenone group treated with OLE (16 mg/kg and 32 mg/kg body weight) showed a beneficial effect (p < 0.001) towards gripping strength. The group treated with 8 mg/kg OLE show less improvement in motor deficits as compared to rotenone intoxicated group (p < 0.05)(Fig. 1B).

The Catalepsy test was performed to check the muscle stiffness. An increase in muscle stiffness was observed (p < 0.001) in rotenone-induced mice compared to control. The holding time in the treatment group (16 mg/kg and 32 mg/kg body weight) mice was significantly lower (p < 0.001) than rotenone intoxicated group. The observation of 8 mg/kg OLE with rotenone group was relatively less significant (p < 0.05) (Fig. 1C). All data are expressed in terms of mean ± SEM.

### Biochemical analysis

#### *OLE reduces the lipid peroxidation in rotenone intoxicated mice model*

**Figure 2:**
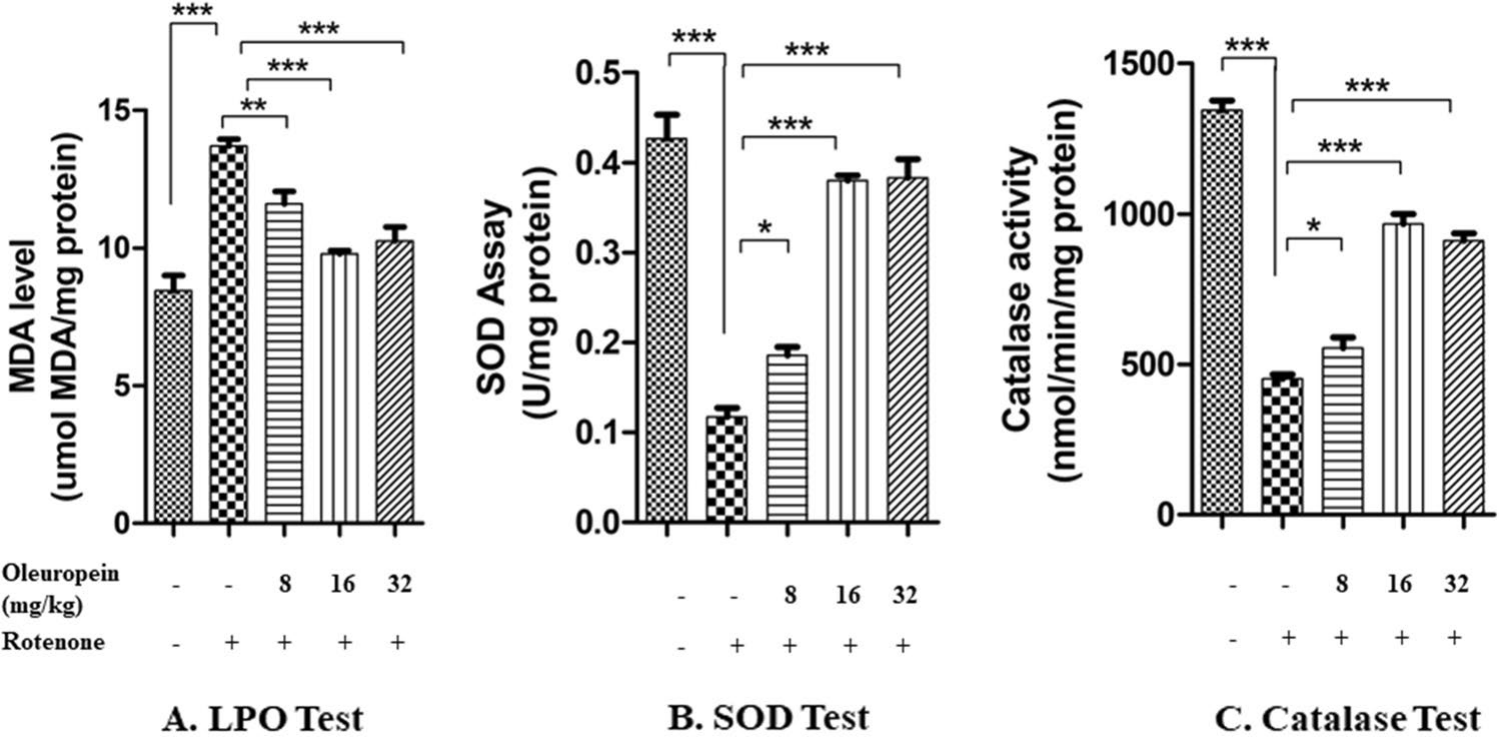
In LPO test, MDA level was elevated in rotenone group as compared to control (***p < 0.001) and decreased in case of OLE treated groups notably in 16 and 32 mg/kg bwt. The SOD and Catalase activity was reduced in case of rotenone than control while in OLE treatment (16 and 32 mg/kg bwt), the SOD and Catalase activity was significantly increased. In all these three biochemical parameters, the OLE doses of 8 mg/kg bwt. showed a less significant effect. (*p < 0.05).The mean ± SEM (n = 6) is used to depict the values.

The high level of MDA was observed in rotenone group as compared to control mice. The group treated with OLE of 16 mg/kg and 32 mg/kg bwt showed the significant decrease (p < 0.001) in level of MDA. When three different dosages of OLE, (8 mg/kg, 16 mg/kg and 32 mg/kg bwt) were given to the rotenone-induced mice, 16 mg/kg and 32 mg/kg body weight were shown to be more effective than the 8 mg/kg body weight group (p < 0.05) (Fig. 2A).

#### *OLE influences the activity of antioxidants*

The results showed that the activity of SOD enzyme was reduced (p < 0.001) in rotenone administrated mice than control group. Rotenone treated with OLE group (specifically in 16 mg/kg and 32 mg/kg body weight) showed significant increase in the activity of SOD (p < 0.001). Likewise, the level of catalase enzyme was significantly reduced (p < 0.001) in rotenone induced mice in comparison with control group and optimum catalase activity was seen in groups of 16 mg/kg and 32 mg/kg OLE (p < 0.001) (Fig. 2B,C).

### Immunohistochemistry analysis

#### *OLE protects loss of SNpc DA neurons from rotenone-induced PD mice and also reduces the loss of striatal DA at nerve terminals*

**Figure 3:**
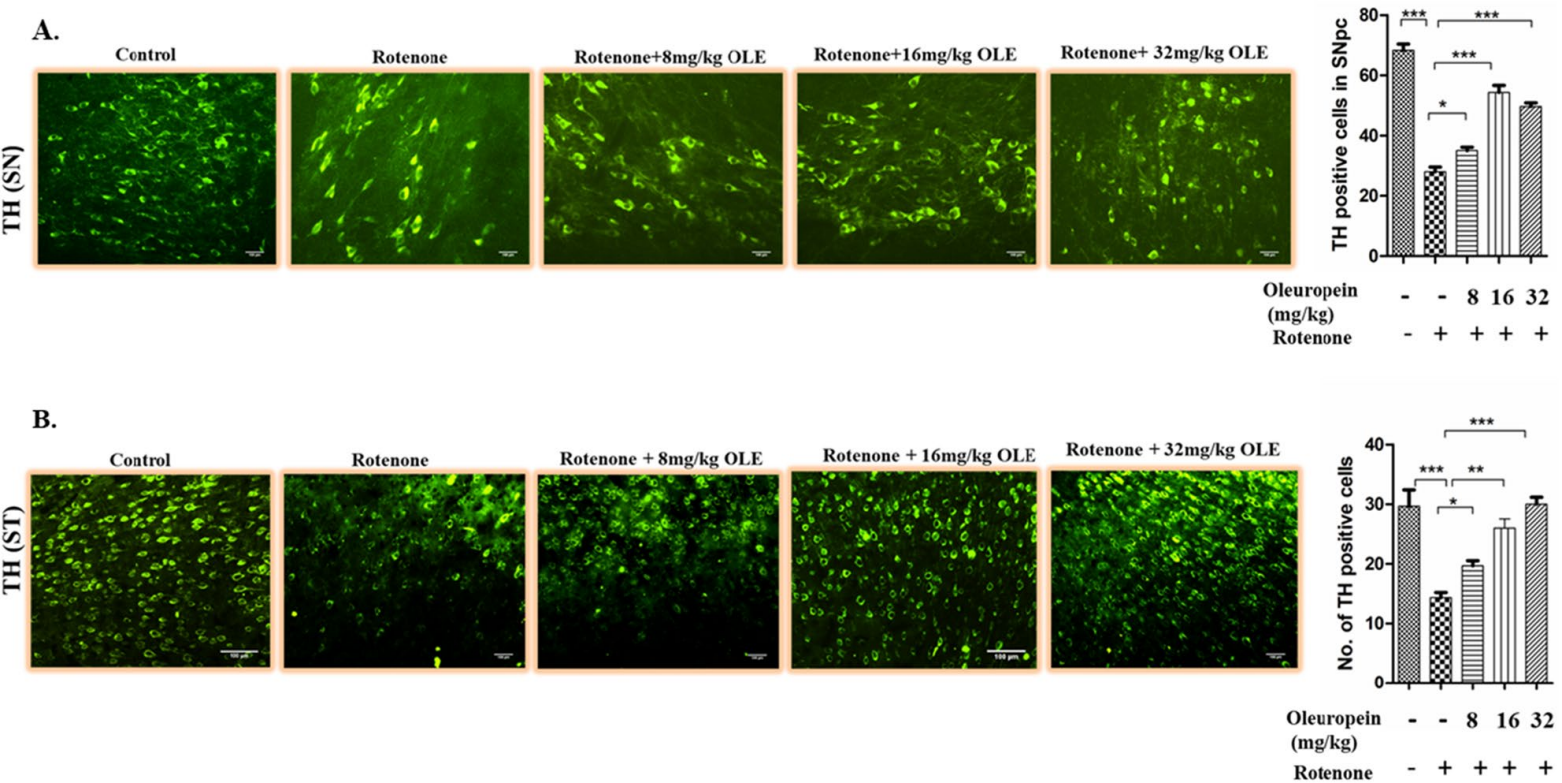
The immunoreactivity of TH in the SN and ST of various experimental groups was examined using immunohistochemistry under the florescence microscope (20×). Rotenone intoxicated group showed less immunoreactivity than control (p < 0.001) in SN and ST. While, increased immunoreactivity of TH was observed in case of OLE treated group (16 and 32 mg/kg bwt) in SN (p < 0.001) and ST (p < 0.01). In all these three biochemical parameters, the OLE doses of 8 mg/kg bwt. showed a less significant effect. (*p < 0.05). Values are expressed as mean ± SEM (n = 6). ***p < 0.001, and *p < 0.05. *SEM* Standard error of mean, *OLE* Oleuropein, *SN* Substantia nigra, *TH* Tyrosine hydroxylase.

The expression of TH in DA neurons in the SN and Striatum (ST) areas of the mouse brain was studied using immunostaining with significant findings between the rotenone groups and OLE treated mice viz. 8 mg/kg, 16 mg/kg and 32 mg/kg body weight. In SN region, rotenone intoxication significantly reduced (p < 0.001) the immunoreactivity of TH when compared to control group. The most significant results were obtained in 16 mg/kg and 32 mg/kg bwt (p < 0.001 and p < 0.001) as compared to 8 mg/kg bwt (p < 0.05) (Fig. 3A). Similarly in ST, rotenone administration cause decrease (p < 0.001) in immunoreactivity than control group whereas, OLE with doses 16 mg/kg and 32 mg/kg bwt (p < 0.01 and p < 0.001) showed increase in immunoreactivity than the PD group whereas, OLE with 8 mg/kg bwt had less significant immunoreactivity (Fig. 3B).

Although both 16 mg/kg and 32 mg/kg bwt doses of OLE were found to be significant, nevertheless, the minimum effective dose of 16 mg/kg bwt was taken for further study.

## Effect of OLE on neuronal survival in rotenone-intoxicated parkinsonian mouse model

### Result 2

#### *OLE alleviates the mitochondrial ETC chain dysfunction in rotenone-induced PD mice*

**Figure 4:**
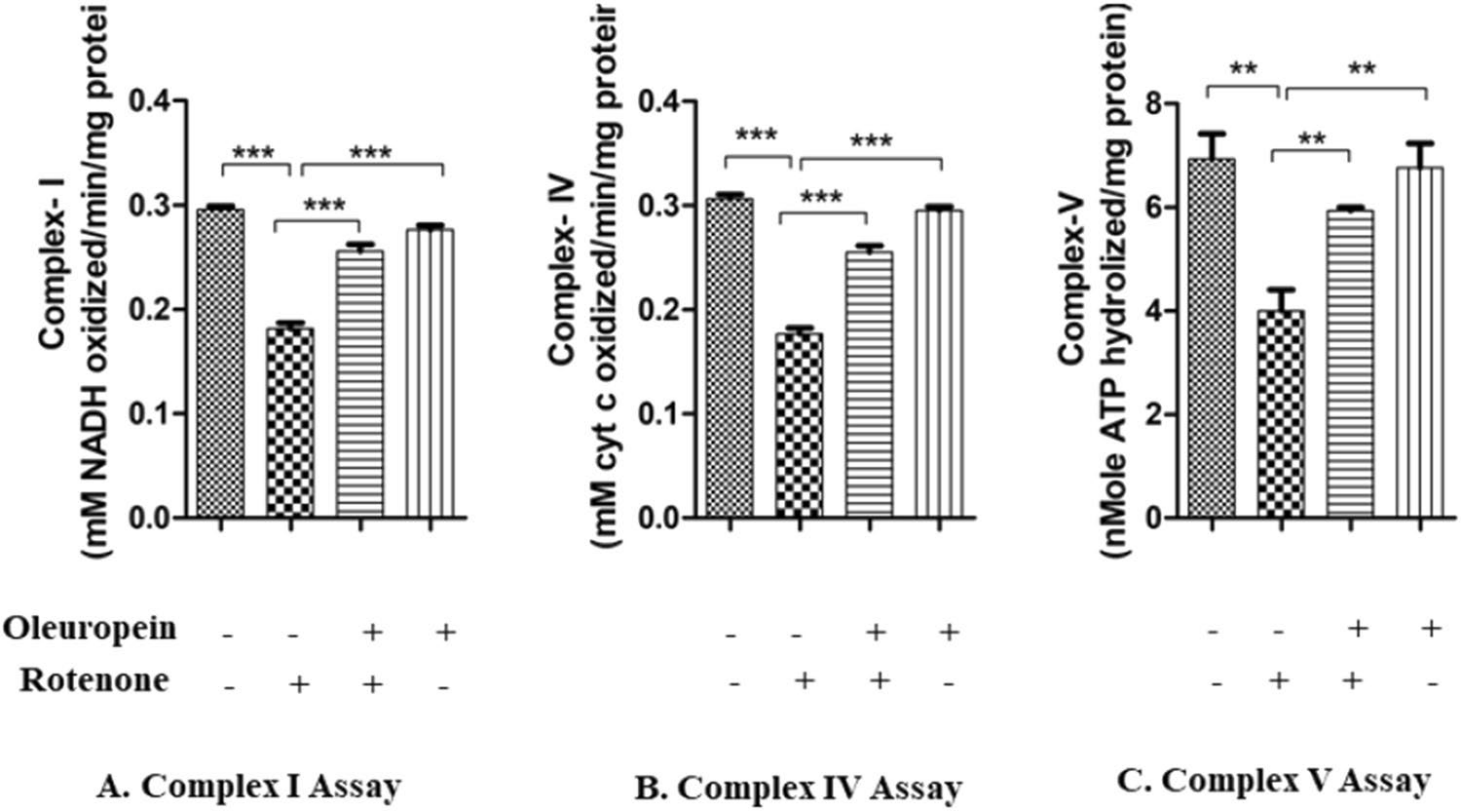
Effect of OLE on mitochondrial complex activity of I, IV and V was observed. In Rotenone-intoxicated mouse brains, the complex activity of I, IV and V was decreased than control whereas in OLE treatment group, significantly increased mitochondrial complexes activity of I, IV and V was observed. The results are provided as mean SEM (n = 5). **p < 0.01, and ***p < 0.001. The mean ± SEM (n = 6) is used to depict the values.

Results showed that OLE had an influence on the mitochondrial dysfunction produced by rotenone intoxication. In our investigation, we found that Rotenone along with inhibiting complex I also inhibits the activity of other ETS complexes. Mechanistically, in comparison to rotenone-intoxicated PD mice, OLE treated group showed reduced mitochondrial impairment and enhanced respiratory chain Complex I (p < 0.001; Fig. 4A), IV (p < 0.001; Fig. 4B), and V (p < 0.01; Fig. 4C) activity. There was a substantial decrease in the activity of complexes I (p < 0.001), complex IV (p < 0.001), and complex V (p < 0.01) in rotenone group as compared to control.

#### *OLE reduced the elevated Ca*^2+^* level in nigrostriatal region of PD mice*

**Figure 5:**
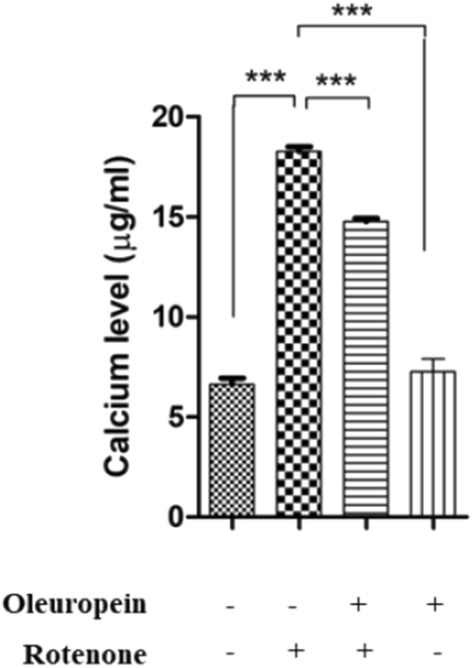
Effect of OLE on Ca^2+^ levels. Rotenone administrated group showed increase in Ca^2+^ levels (***p < 0.001) than control. Additionally in OLE-treated group, significantly reduced Ca^2+^ level was found (***p < 0.001). The results are provided as mean SEM (n = 6),

Ca^2+^levels in the nigrostriatal tissue of PD mice were found to be substantially greater than in control mice (p < 0.001). While OLE treatment led to significant decrease in Ca^2+^ level (p < 0.001) as compared to rotenone-intoxicated groups (Fig. 5).

#### *OLE reduced the apoptotic markers in rotenone-induced PD model*

The impact of OLE on proapoptotic (Bax, Caspase3) and antiapoptotic factor (Bcl-2) were examined in PD mice (Fig. 9A). The Bax/Bcl-2 ratio in Rotenone group was considerably higher (p < 0.01) than the control group, and lower in the OLE-treated PD mice (p < 0.01; Fig. 9B), demonstrating the antiapoptotic function of OLE in the parkinsonian mouse model. Similarly, cleaved caspase-3 also get upregulated which supports the increased (p < 0.001) expression of caspase-3 in case of rotenone group as compared to control whereas in OLE administrated group, it was significantly reduced (p < 0.01; Fig. 9C).

#### *OLE enhanced the interaction of BDNF and TrkB by alleviating the aggregation of α-synuclein*

**Figure 6:**
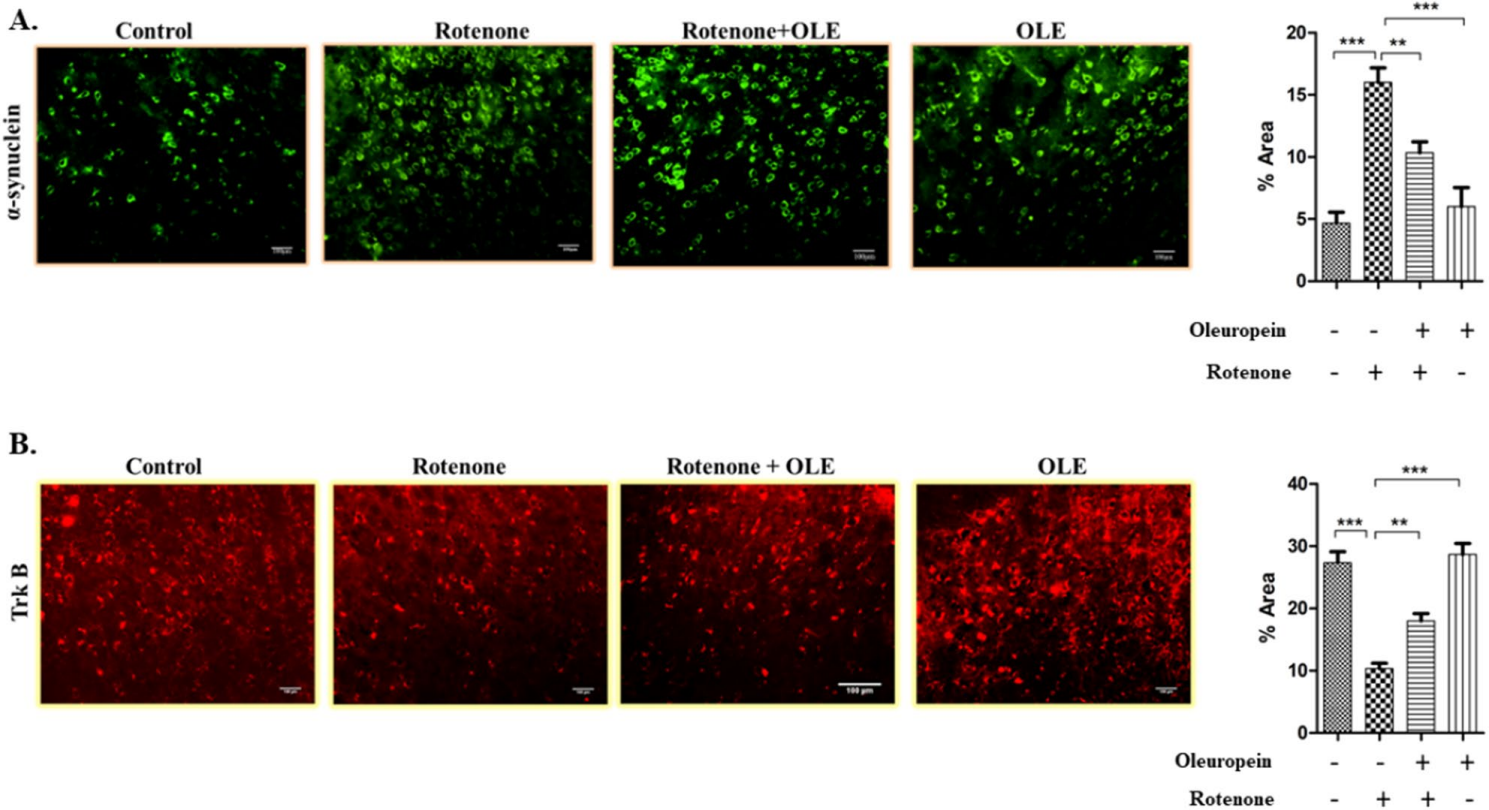
The immunohistochemical staining of (**A**) α- synuclein and (**B**) Trk B. The increase in expression of α-synuclein in rotenone group (***p < 0.001) while neuroprotection of OLE significantly decreased the expression of α-synuclein (**p < 0.01). Similarly, Trk B expression was reduced upon rotenone intoxication (***p < 0.001) whereas, OLE significantly upregulated the expression of Trk B in OLE treated group (**p < 0.01). The one-way ANOVA was used to analyse the data, followed by the Newman–Keuls test. The mean ± SEM (n = 6) is used to depict the values.

**Figure 7:**
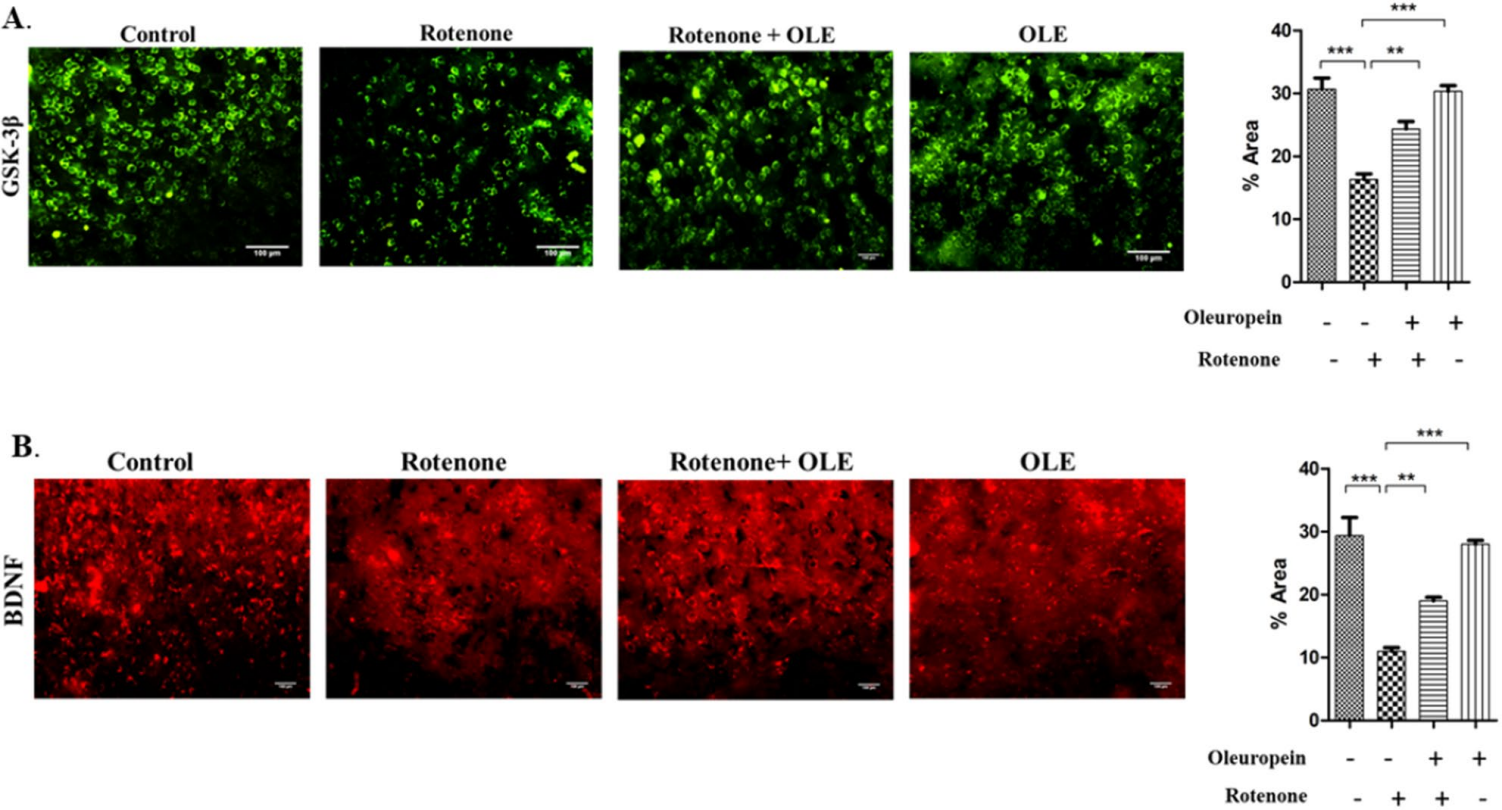
In comparison with the control, immunohistochemical labelling of (**A**) p-GSK-3β and (**B**) BDNF in SN region different experimental groups revealed a reduction in expression in the rotenone group (***p < 0.001), whereas OLE treatment dramatically increased p-GSK-3β and BDNF expression(**p < 0.01). The florescent image was analysed by software image J at 20× magnification. The one-way ANOVA was used to analyse the data, followed by the Newman–Keuls test. The mean ± SEM (n = 6) is used to depict the values.

**Figure 8:**
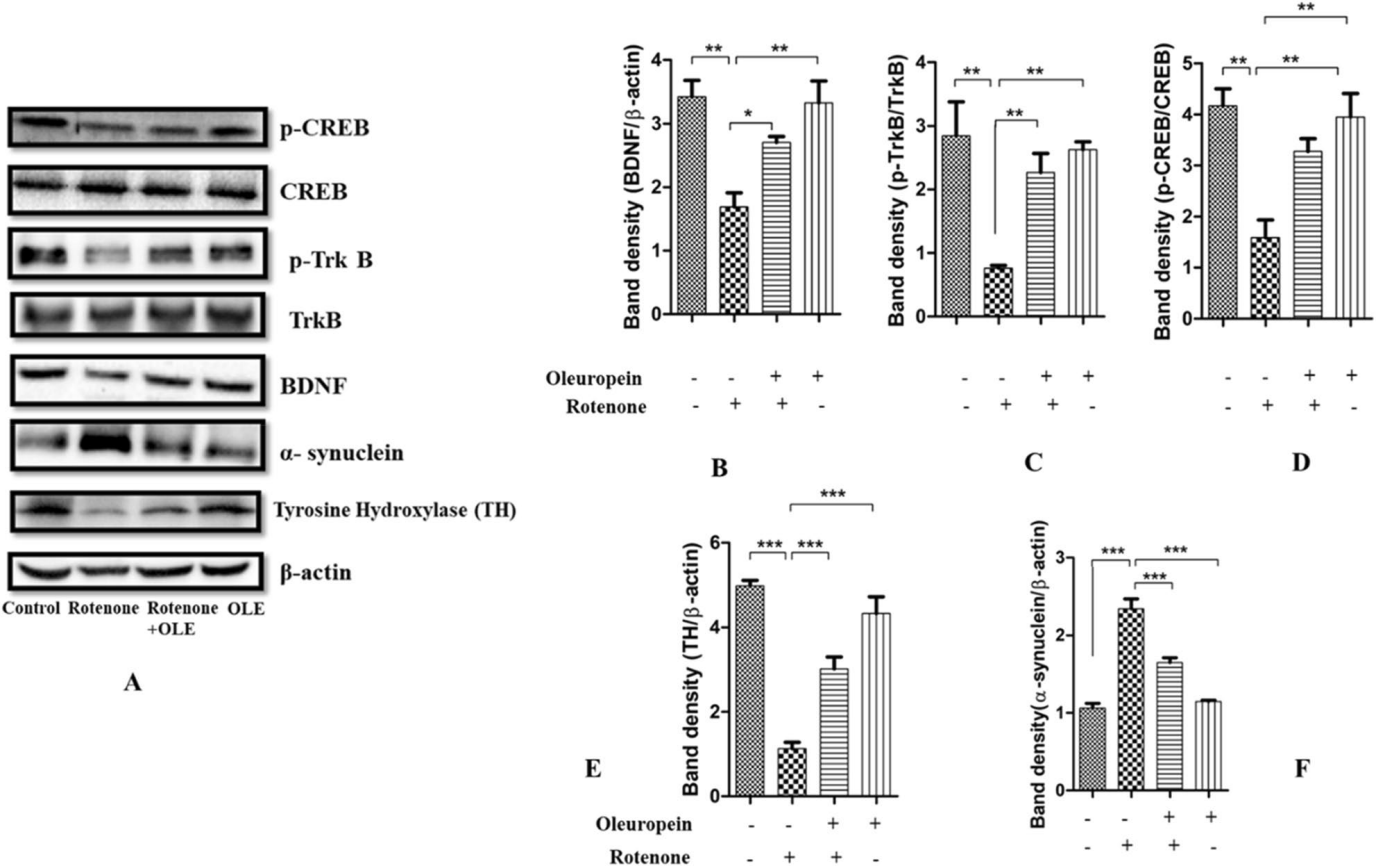
(**A**–**F**) This figure shows the relative expression of α-synuclein, TH, p-Trk B, BDNF, p-CREB, in which β-actin acts as a control (**A**). The lower expression of BDNF (**B**), p-Trk B (**C**), CREB (**D**), TH (**E**) was observed in case of rotenone-intoxicated mice (***p < 0.001) (**p < 0.01) respectively. while OLE administered group showed significantly increased expression of TH, CREB, BDNF and p-Trk B and BDNF (***p < 0.001) (**p < 0.01) respectively. Aggregation of α-synuclein (**F**) was found to be more in rotenone group (***p < 0.001) than control whereas OLE-treated group showed lower aggregation of α-synuclein (***p < 0.001). The one-way ANOVA was used to analyse the data, followed by the Newman–Keuls test. The mean ± SEM (n = 6) is used to depict the values.

It was shown that α-synuclein suppresses BDNF expression and interacts directly with TrkB receptors (as explained above) (Fig. 8A). Hence, decrease in the ratio of p-TrkB/TrkB was seen in rotenone (p < 0.01) as compared to control whereas after treatment with OLE, it was significantly enhanced (p < 0.01; Fig. 8C) among parkinsonian mice group. Similarly, less immunoreactivity of TrkB in SNpc region was seen in rotenone administrated group (p < 0.001) than control group while OLE mitigates the TrkB expression (p < 0.01; Fig. 6B). Moreover, the decrease in expression and immunoreactivity of BDNF was observed in PD mice (p < 0.001) (Fig. 7B; p < 0.001; Fig. 8B) respectively and there was prominent elevation in expression and immunoreactivity of BDNF in OLE treated group (p < 0.01). In terms of α-synuclein expression, the rotenone group exhibited substantially more aggregation than the control group (p < 0.001) and significantly reduced aggregation was seen in case of OLE treated group (p < 0.001; Fig. 8F). In addition, increase in immunoreactivity of α-synuclein was found in SNpc region of rotenone-induced mice (p < 0.001) in comparison with control, whereas treatment with OLE showed significant reduction in the immunoreactivity (p < 0.01; Fig. 6A).

#### Effect of OLE on rate-limiting enzyme TH in PD mice

Reduced expression of TH was seen in nigrostriatal region of rotenone intoxicated group (p < 0.001). While it was highly upregulated in OLE treated group (p < 0.001) in comparison with PD mice (Fig. 8E). These finding suggests that OLE helps in regulating the expression of rate limiting enzyme in parkinsonian mice model.

#### OLE ameliorates the expression of CREB and phosphorylation of kinases, Akt and GSK-3β

**Figure 9:**
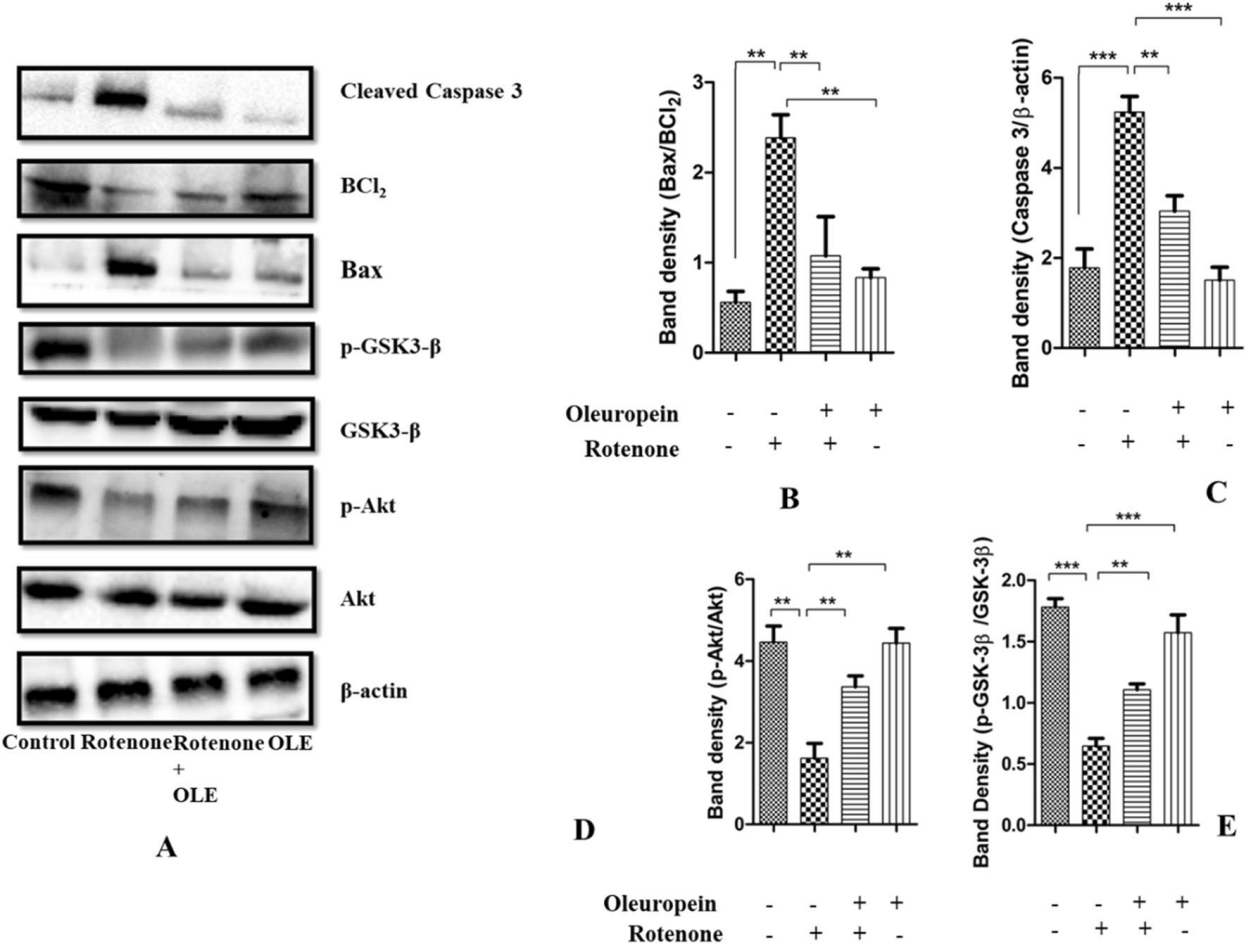
(**A**–**E**) Using the Western blotting method and protein densitometry analysis, the relative expression of Bax, Caspase-3, Bcl-2, p-Akt and p-GSK-3β was examined in the SN of mice (**A**). OLE inhibited the increase in ratio of Bax/BCl_2_ (**B**) and decrease in the expression of cleaved caspase 3 (**C**). Additionally, phosphorylation of Akt (**D**) and GSK3β (**E**) was increased by OLE. The one-way ANOVA was used to analyse the data, followed by the Newman–Keuls test. The mean ± SEM (n = 6) is used to depict the values (***p < 0.001 and **p < 0.01).

OLE promoted the neuronal survival gene by modulating the multitarget kinases, Akt and GSK-3β dysregulation. Rotenone group had a lower ratio of p-Akt/Akt (p < 0.01) than control, and the treatment group had a significantly higher p-Akt/Akt ratio (p < 0.01; Fig. 9D). Recent research has connected GSK-3β to intrinsic apoptosis in mitochondria and the activation of CREB. The ratio of p-GSK-3β/GSK-3β was declined in rotenone intoxication (p < 0.001) whereas it was highly ameliorated in OLE administrated group (p < 0.01) (Fig. 9E). Similarly, IHC was done to further support the findings, and the results showed a significant drop (p < 0.001) in GSK-3β phosphorylation among PD group as compared to the control group while the effect of OLE treatment showed increase in GSK-3β phosphorylation (p < 0.01; Fig. 7A). In addition, OLE induced the phosphorylation of CREB by increasing the ratio of these kinases. Hence, the ratio of p-CREB/CREB was increased significantly in OLE administrated group (p < 0.01) and decreased in diseased condition (p < 0.01; Fig. 8D).

## Discussion

**Figure 10:**
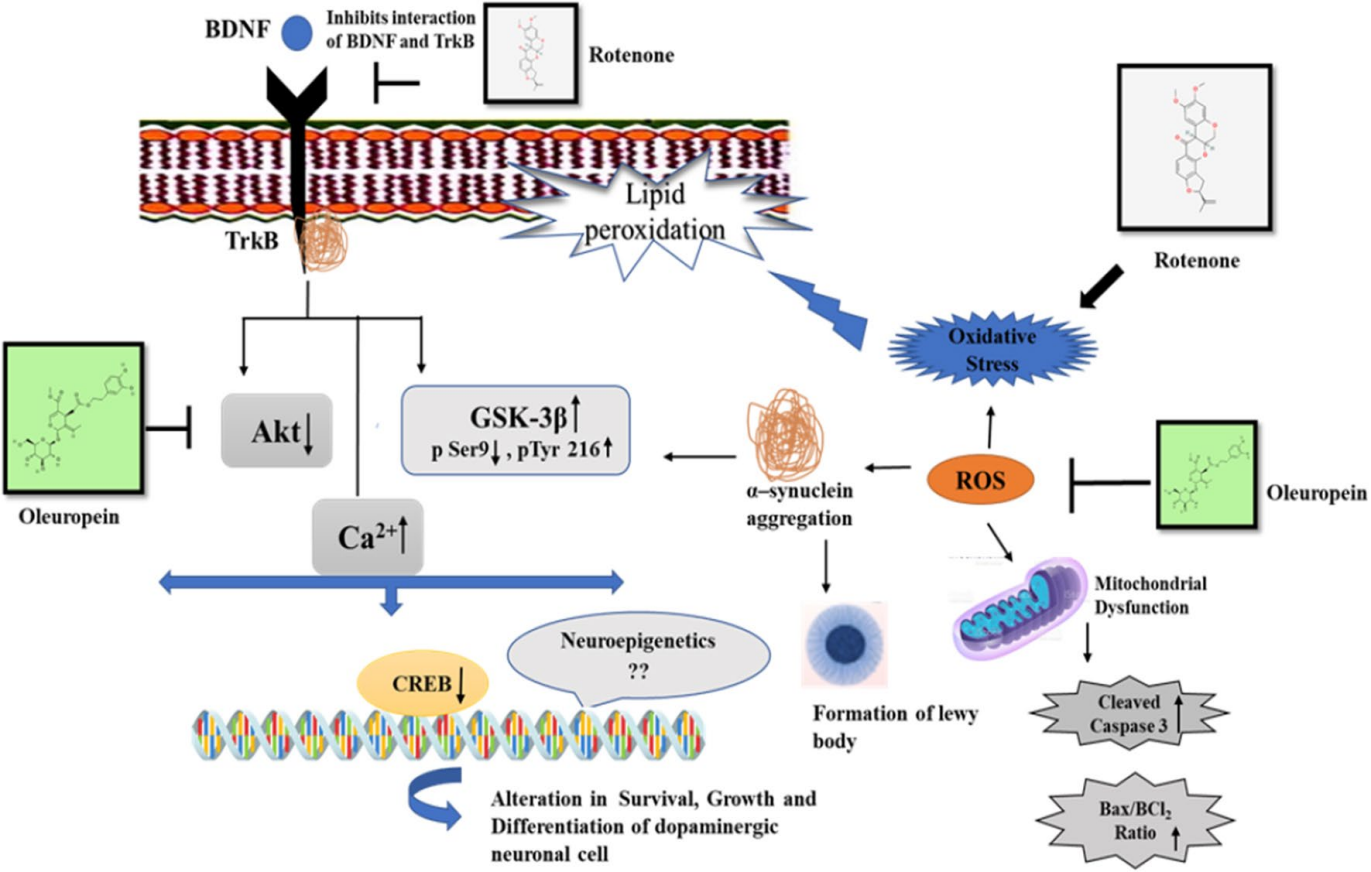
The mechanism of neuroprotection of Oleuropein in rotenone induced PD mouse model.

The survival of neurons is maintained by a complex interconnected network of signalling cascade that can be disrupted by a variety of cellular stressors. A change in the balance of signalling pathways in response to stress or illness can have dramatic ramifications on growth and differentiation of neuronal cells^37,38^. This causes compromised mitochondrial structure, energy metabolism, and nuclear integrity^39,40^. These processes are primarily regulated by one of the most important neurotrophic factors such as BDNF, which is important for dopaminergic neuronal survival, plasticity, and differentiation^41^. PD is a chronic and progressive disease; its symptoms worsen with time. Current PD medications are symptomatic, necessitating the finding of potent neuroprotectants with fewer side effects^42^. We investigated the efficacy of OLE in rotenone model of Parkinson's disease and found that it reduced the pathological and phenotypic characteristic of PD. OLE promotes the survival, growth and differentiation of dopaminergic neurons through its anti-oxidative and anti-aggregative effects^43^. Our study, reveals the dynamic changes in the cascade of signalling proteins which are directly related to the degeneration of dopaminergic neurons through Akt/CREB/BDNF pathway. We have also observed the reduced ROS production, mitochondrial dysfunction and apoptosis by OLE in Rotenone induced mice model as shown in Fig. 10.

The findings of our study are consistent with those of previous investigations which have indicated that OLE exhibits a neuroprotective role against chronic stress, induced in parkinsonian mice model. In dose standardisation study we evaluated the effect of different concentration of OLE i.e. 8, 16 and 32 mg/kg bwt on rotenone induced mouse model of PD.

It was observed that mice intoxicated with rotenone showed motor impairment, including prolonged narrow beam walking, reduced hanging time and catalepsy duration. While, administration of 16 and 32 mg/kg bwt of OLE, results in the reduction of latency time in the narrow beam test as well as an increase in hanging time and catalepsy time in PD mouse. Our findings suggests that OLE treatment significantly improved postural instability, muscular strength and motor impairment induced by rotenone intoxication.

Reduced activity of some major antioxidant enzymes such as SOD and catalase in rotenone-treated cells has been reported^44,45^. In our study, we have also observed the decrease in enzymatic activity of SOD and catalase with rotenone intoxication due to increase ROS generation. While dose of 16 and 32 mg/kg bwt OLE administration, increases the enzymatic activity by scavenging ROS. This could be due the formation of intra molecular hydrogen bonding of OLE with free radicals^46^. Stressful environment produces free radicals, which then interact with free oxygen molecules on membrane lipids to produce peroxy radicals, which are responsible for lipid peroxidation^47^. Our research confirms that Rotenone-intoxicated mice had higher MDA levels in the nigrostriatal area, as reported by other^48^ whereas OLE treatment results in decrease in MDA level.

Since TH is a rate limiting enzyme in the synthesis of dopamine^49^, our study also deals with the observation of TH immunoreactivity in SN and ST region. Due to rotenone intoxication, reduced immunoreactivity of TH was found whereas, 16 and 32 mg/kg bwt of OLE showed a significant increase in expression of TH. OLE reversed the loss of TH expression and maintained the TH integrity of nerve terminals. On the basis of neurobehavioral analyses, metabolic data, and TH immunoreactivity it has been observed that 16 and 32 mg/kg bwt dose of OLE are more efficacious than the 8 mg/kg bwt dose of OLE.

Rotenone reduced the production of ATP in dopaminergic neurons to a higher extent than non-dopaminergic neurons by inhibiting complex I^40^. Mitochondrial biogenesis is also influenced by signalling molecules and neurotrophic substances such as BDNF^50^. The effect of BDNF, GDNF, and NGF on oxidative metabolism in mouse brain mitochondria has been investigated in vitro^51,52^. In PD, blocking GSK-3β activation protects dopaminergic neurons against rotenone-induced toxicity by preventing apoptosis caused by complex I inhibition^53^. Our observation reinforced the previous evidence by showing the reduced activities of ETS complexes I, complex IV, and complex V due to mitochondrial dysfunction. Aggregation of α-synuclein and increase in oxidative stress must have contributed to the reduced complex activity. This can be linked to the increased GSK3β activation and Ca^2+^ levels in the cytoplasm. It is therefore hypothesised that the altered activities of the mitochondrial complexes caused by rotenone, prevented the transport of electron between the complexes, which was reduced by the administration of OLE.

TrkB has a high affinity for BDNF, which aids in the survival of Nissl-stained neuronal cells and dopaminergic neurons, as well as preserves dopaminergic connections to the ST^54^. Research has shown that TrkB expression was dramatically decreased in MPTP-induced C57/BL6 mice models of PD^55^. α-Synuclein aggregates interacts directly with TrkB receptor which hinders the interaction of BDNF and TrkB. This leads to reduced neurotrophic activities, making DA neurons more vulnerable to degeneration^56^. Hence, α-synuclein-mediated upregulation of TrkB ubiquitination, hindered TrkB axonal trafficking and decreased TrkB protein level^57^. The current study validated the previous concept by demonstrating the decrease in the expression of BDNF and TrkB in the rotenone intoxicated mice model due to the accumulation of α-synuclein. OLE has significantly induced BDNF-TrkB interaction and helps in suppression of α-synuclein pathology. This might be due to the conformational change in TrkB receptor induced by OLE which facilitates the binding of BDNF and TrkB, showing its neuroprotective role.

In several studies, Akt has been found to play a role in cellular signal transduction, as it is activated after binding of TrkB and BDNF^58,59^. GSK-3β and α-synuclein interaction results in the disruption of BDNF signalling, which is one of the complex pathways in PD neuropathology^60,61^. According to Golpich et al., Akt can decrease GSK-3β activity by phosphorylating ser9 of GSK-3β which downregulate the series of programmed cell death^62^. Furthermore, aberrant GSK-3β regulation might result in PD pathophysiological symptoms^63^. GSK-3β inhibitors have been found to increase both BDNF mRNA and protein in cultured cortical neurons, suggesting that GSK-3β activity has a direct effect on neuronal BDNF level^64,65^. In accordance with previous research, our findings showed the reduction in BDNF and TrkB expression. The p-Akt/Akt ratio and the p-GSK3β/GSK3β ratio were both lowered in the rotenone group, whereas they were significantly enhanced in the OLE administered group. The immunohistochemistry technique also proved the previous finding and shows reduced expression of p-GSK3β in case of rotenone while OLE increased the expression of p-GSK3β, exhibiting their neuro-survival effect. The BDNF-TrkB interaction and the binding of CREB to the BDNF promoter was seen in the aforementioned data, suggesting that targeting the neuronal survival pathway might be more advantageous for the treatment strategy in the case of PD.

Rangasamy et al. have shown that CREB modulates the expression of TH gene by binding to its promoter and thus the expression of both CREB and TH was shown to be downregulated in PD mouse model^66,67^. We have also observed the lower expression of p-CREB and TH protein in nigrostriatal region of rotenone induced mouse and higher expression in OLE treated group through Western blotting analysis.

Additionally, the lower levels of CREB phosphorylation may be caused by the inactivation of upstream signalling proteins, resulting in reduced expression of BDNF in the rotenone group. Phosphorylation of CREB and BDNF was significantly reduced by OLE which verified the earlier evidences. The results of our experiment support that CREB is an important modulator of neurotrophic response and neurons have developed several neurotrophic signalling pathways to CREB.

It was observed that mitochondrial dysfunction and ROS overproduction leads to the disturbance in Ca^2+^ homeostasis in case of PD^68^. Ca^2+^, a key second messenger, is hypothesised to modulate some downstream proteins of BDNF in a rapid and localized way^69^. Ca^2+^ binding to α-synuclein speeds up the production of α-synuclein fibrils in vitro, perhaps contributing to PD etiology^70,71^. In the present study, rotenone administration causes a rise in nigrostriatal Ca^2+^ level, and OLE treatment results in a significant decrease in cytosolic Ca^2+^ level, suggesting a neuroprotective role of OLE to diminish the progression of PD.

Aggregation of α-synuclein has also been found to increase the Bax/Bcl-2 ratio in several in vitro and in vivo studies of PD^72,73^. However, mitochondrial dysfunction results in upregulation of proapoptotic factor which triggers the activation of caspase 3, leading to the neuronal cell death^74^. Bcl-2 family members can also be activated by BDNF as reported in MPP^+^-induced neuronal cell line SH-SY5Y cells and mouse models^75^. Similarly, our Western blotting experiment revealed a decrease in the ratio of Bax/Bcl-2 and cleaved Caspase3 in the rotenone induced mouse model due to abrupt increase in ROS level that resulted in uncontrolled series of apoptosis. However, OLE treatment has rescued DA neurons from rotenone-induced toxicity by decreasing the ratio of such pro-apoptotic factors markedly.

Therefore, our finding suggests that the regulation of growth and differentiation of neuron is involved in the origin of PD pathogenesis. Hence, it is required to target neuronal survival pathway from the therapeutic point of view in the treatment of PD. Since PD is primarily sporadic, it will be interesting to study these cellular pathways at the epigenetic level so that genetic and environmental factor, both can be explored for deeper understanding of the disease pathogenesis.

## Concluding remarks

Our research suggests that OLE can provide potent neuro-survival effect against the key factors of neurodegeneration in PD, like oxidative stress, α-synuclein upregulation, altered Ca^2+^ level, mitochondrial dysfunction, downregulation of neurotrophic factors and apoptosis by regulating BDNF/Akt/CREB signalling. OLE should be studied further at the epigenetic and pre-clinical level as a prospective pharmacological option for amelioration of neurodegeneration in PD.

## Dose chase experiment for the analysis of neuroprotective action of OLE

### Methods

#### Ethical approval

The institutional animal ethics committee of BHU in Varanasi, India, accepted this study. The experiments were carried out in accordance with the institutional ethical standards, and they were approved by the institutional animal ethics committees (IAECs) of the laboratory animal research at Banaras Hindu University in India (IAECs Approval Reference No. BHU/DoZ/IAEC/2021-2022/031). Animal experiment design, execution, and reporting all according to the ARRIVE guideline. The number of mice used was kept to a minimum. Every attempt was made to reduce animal pain.

#### Experimental animals

Eight to ten-week-old male Swiss albino mice (25 ± 5 g) were facilitated by the Institute of Medical Sciences at Banaras Hindu University in India. Mice were acclimatised to laboratory settings with constant light–dark cycles of 12 h for 7 days prior to the commencement of the experiment. All animal experimental protocols were approved by the Animal Ethics Committee of Banaras Hindu University in Varanasi, India.

#### Experimental designs

The first group had employed as control and the second group was injected with rotenone. The vehicle for the control group was 0.9% normal saline. The third, fourth, and fifth groups was initially administered with intraperitoneal doses of OLE i.e. 8 mg/kg, 16 mg/kg and 32 mg/kg body weight respectively for 7 days and later was simultaneously administered with rotenone for 35 days**.** The solubility of OLE was in 0.9% normal saline and rotenone was diluted in sunflower oil to a final concentration of 2 mg/ml after being dissolved in chloroform at a 50× stock solution. Following the completion of dosage, behavioural parameters was assessed and the mice from each group was sacrificed and their brains was collected by decapitation for biochemical tests and further experimental work.

#### Reagents and antibodies

Oleuropein, Rotenone, acetic acid, Bradford reagent (Himedia), EGTA (Ethylenediamine tetraacetic acid), EDTA (ethylene glycol-bis(β-aminoethyl ether)-*N*,*N*,*N*′,*N*′-tetraacetic acid), Tris-buffer, sodium dodecyl sulphate (SDS), paraformaldehyde, sodium chloride, sodium hydroxide, disodium hydrogen phosphate, sodium dihydrogen phosphate, bovine serum albumin (BSA), ammonium chloride, potassium chloride and reduced nicotinamide adenine dinucleotide phosphate (NADPH)were obtained from Sisco Research Laboratories (SRL, Mumbai, India). Potassium dichromate and hydrogen peroxide (H_2_O_2_) were purchased from Merck (Darmstadt, Germany). Thiobarbituric acid (TBA), 1,4-diazabicyclo 2.2.2 octane (DABCO), and Griess reagent were obtained from HiMedia (Mumbai, India). Lobachemie, India provided the paraformaldehyde and sodium nitrite. Normal goat serum (NGS) was purchased from Sigma–Aldrich (St. Louis, MO, United States). Primary antibody for Akt (Cat. Ab8805), p-Akt (Cat. Ab81283), Bcl-2 (Cat. GR99542-4) and Ca^2+^ detection kit were acquired from Abcam Life Science, Biogenuix Medsystems Pvt. Ltd. (New Delhi, India), and primary antibodies for p-GSK-3β (Cat. SC81462), p-GSK-3β (Cat. SC373800), TH (Cat. SC25269), α-Synuclein (Cat. SC12767), β-actin (Cat. SC47778), and Bax (Cat. SC6236) were purchased from Santa Cruz Biotechnology (Santa Cruz, CA, United States). BDNF (Cat. PA5-95183), CREB (PA1-850) and Caspase-3 (Cat. PA5-77887) and TrkB (Cat.OST00127W) antibody were purchased from Invitrogen (Thermofisher Scientific). p-TrkB (Cat. ABN1381) antibody was purchased from merkmillipore (Sigma Aldrich). p-CREB (E-AB-20849) was brought from Elabscience.

#### Behavioural assessments

The following behavioural tests were carried out to assess the motor impairment in rotenone-induced Parkinsonian mice. The mice were trained for three days before to the commencement of the experiment. The motor research employed a variety of tests, including narrow beam walking tests, hanging and catalepsy test.

#### Narrow beam walking test

The test was conducted to evaluate motor coordination in mice, which is necessary for maintaining balance while travelling on a narrow beam. The test was carried out to assess the motor coordination of mice, which is essential to maintain balance while travelling on a narrow beam. Animals were originally upskilled to walk on a fixed thin wooden beam 100 cm above the flat surface (L100 cm W1 cm). The time of mice to traverse the beam was noticed, and the experiment was repeated three times^76^.

#### Catalepsy test

In a standard catalepsy test, mice were placed in an odd position and the time it takes to correct it was recorded and that time was used to gauge the severity of catalepsy. Mice were carefully placed with their forelimbs on the bar and their hindlimbs on the floor. When a mouse lifted its hind limbs off a wooden platform, the degree of catalepsy was measured (3 cm). The mice were acclimated for 3 min, and the test was terminated if the delay exceeded 180 s^77^.

#### Wire hanging test

This neuromuscular behaviour test was used to check the grip strength of the mice. In this test, mice were positioned on a top of wire cage lid and the cage had been inverted. The mice were then hung upside down on the grid until they lost their grasp and fell. The length of time spent suspended was recorded, and the experiment was repeated three times^78,79^.

## Biochemical assessments

After numerous behavioural indicators were examined, mice were sacrificed by cervical dislocation followed by decapitation with minimal pain. The brains of mice were removed and placed on ice to be used later. Mice brains were dissected under ice cold conditions for biochemical experiments, and SN and striatal tissue were separated and maintained at − 20 °C until the procedures were finished^80^. The tissue was then homogenised in KCl buffer (Tris–HCl 10 mM, NaCl 140 mM, KCl 300 mM, ethylenediaminetetraacetic acid 1 mM, Triton-X. To obtain supernatant, the tissue homogenates were centrifuged for 20 min at 4 °C at 12,000*g*.

### Estimation of level of Malondialdehyde (MDA) through lipid peroxidation assay

The level of MDA was measured through LPO test of nigrostriatal tissue as described in earlier study with few modified steps^81,82^. Briefly, First, 10% tissue homogenate was mixed with 10% SDS, followed by 20% acetic acid. The reaction mixture was then introduced in 0.8% TBA and kept in a boiling water bath for an hour. After cooling the test mixture and centrifuging the supernatant, the absorbance was measured at 532 nm against a control (μmols of MDA per mg protein were used to measure lipid peroxidation).

### Estimation of activity of antioxidant enzymes

Inhibition of mitochondrial complexes, especially complex I by rotenone, can cause oxidative stress and ROS production and also results in reduction of activity of antioxidant enzymes. The catalase activity was assessed using a spectrophotometer to measure the rate of breakdown of its substrate hydrogen peroxide^83^. To accomplish this, the nigrostriatal tissue was incubated in a boiling water bath for 10 min. with potassium dichromate and acetic acid (1:3), and the OD was measured at 570 nm. The enzyme activity was calculated in nmoles/min/mg protein. NADH was used as a substrate to measure SOD activity^84^. Nitro blue tetrazolium chloride (NBT) reduction was inhibited by an enzyme source based on the difference between reference and experimental OD of the sample. The protein was also approximated using the enzyme source. Finally, the absorbance was measured against a reagent blank at 560 nm. SOD activity was measured in units per mg of protein.

### Evaluation of TH in SNpc by immunohistochemical staining

#### Tissue processing

Each group of mice was anaesthetized with pentobarbital, then perfused intracardially with 0.9% saline (chilled) and 4% paraformaldehyde (chilled) produced in 0.1 M phosphate buffered saline (PBS), pH 7.4, before being decapitated. Brains were removed and fixed overnight in 10% paraformaldehyde and then swapped three times a day with sucrose solution (10%, 20% and 30%) for three consecutive days at 4 °C, before being cryoprotected in a 30% sucrose solution. A cryomicrotome (Leica, Wetzlar, Germany) was used to cut 15 µm thick coronal slices passing through SNpc^85^. The sections were rinsed three times in 0.01 M PBS (pH 7.4) before being blocked for 1 h with 10% NGS in PBS, 0.3% Triton-X 100, and 1% BSA in phosphate-buffered saline with Triton X-100 (PBST). The sections were subsequently treated with a polyclonal anti-mouse antibody against TH, Tyrosine Hydroxylase (TH) antibody (1:1250 dilution). After that, the brain sections were incubated at room temperature with FITC-conjugated secondary antibodies (for use with anti-mouse primary) (2 h).The images were acquired with a fluorescence microscope (Nikon, Thermo Fisher Scientific), and then further processed using ImageJ software (File Version 1.4.3.67) (NIH, United States).

### Statistical analysis

Graph Pad Prism software (Version 5.01) was used to analyse the data, which included one-way analysis of variance (ANOVA) and the student–Newman–Keuls test. The results are presented as means with standard deviations (SEM). A p-value of 0.05 or below (p < 0.05) was considered statistically significant. At each level, the experiments were repeated three times. For various experimental studies control was used appropriately as per requirements.

### Effect of OLE on neuronal survival in rotenone-intoxicated parkinsonian mouse model

#### Experimental design

Mice were divided into four groups, each with six individuals. Group I served as the control group, receiving only 0.9% normal saline. Rotenone was administered to the second group; OLE and rotenone were given to the third; and OLE (16 mg/kg bwt.) alone was given to the fourth group. For the first week, Group-III was given intraperitoneally with OLE as pre-treatment and further co-treated with the dose of rotenone for 35 days. In earlier experimental designs, the rotenone and OLE administration methods were detailed.

### Mitochondrial assays

#### Isolation of mitochondria

The nigrostriatal area of the midbrain was homogenised using a solution (pH 7.4) containing 225 mM mannitol, 5 mM HEPES, 75 mM sucrose, 1 mM ethylene glycol tetra acetic acid (EGTA), and 1 mg/ml BSA. The mitochondrial pellet was separated from the mouse brain by differential centrifugation^86^. After isolating the nigrostriatal region of brain, the tissue was homogenized with homogenization buffer. The homogenate was then centrifuged for 30 min. at 4 °C at 2000*g*. After centrifugation, the supernatant was centrifuged again at 12,000*g* for 10 min. The mitochondria and synaptosomes were obtained from the pellet produced after the second centrifugation and suspended in homogenising buffer containing digitonin (0.02%). To get a crude mitochondrial fraction, the mixture was again centrifuged for 10 min at 12,000*g*. Mitochondrial pellets was then rinsed twice with homogenising buffer without BSA or EGTA, and the fraction was resuspended in phosphate buffer (50 mM, pH 7.4). The Bradford test was used to determine the concentration of proteins in all of the samples. The experiments were carried out using 20 g proteins within 24 h after mitochondrial isolation.

#### Complex I assay

In our investigation, the testing of complexes I was carried out using the technique of Stojakovic et al.^87^. The catalytic oxidation of NADH to NAD^+^ reduced the cytochrome *c* in this experiment. The reaction mixture was made up of NADH (6 mM in 2 mM glycylglycine buffer), glycylglycine buffer (0.2 M, pH 8.5), and cytochrome *c* (10.5 mM). It was then mixed with 20 g mitochondrial protein and the absorbance was measured at 550 nm for 2 min. At 340 nm, NADH has an extinction coefficient of 6.22/mM/cm. The activity of the enzymes was measured in nmol NADH oxidized/min/mg protein.

#### Complex IV assay

The oxidation of reduced cytochrome c at 550 nm was used to evaluate Complex IV activity^88^. Potassium ferricyanide, 10 mM phosphate buffer (pH 7.4), reduced cytochrome *c*, and 20 g mitochondrial protein were added in the reaction mixture. A few crystals of sodium borohydride were added to a solution of oxidised cytochrome *c* (10 mg/ml) to produce reduced cytochrome *c*. The complex IV activity was measured in nmol cytochrome *c* oxidized/min/mg of protein after observing the change in absorbance for around 3 min.

#### Complex V assay

The quantity of inorganic phosphorus released during ATP to ADP hydrolysis was determined in this test. Sarafian’s approach was used to test mitochondrial ATPase^89^. The mitochondrial protein was incubated for 5 min at 30 °C in ATPase buffer (5 mM ATP, 2 mM MgCl_2_, and 50 mM Tris HCl, pH 8.5). The reaction mixture was spun at 3000*g* for 10 min. after 10% TCA was added to it. The results were expressed as nmol inorganic phosphate (Pi) liberated per mg protein per minute.

### Determination of Ca^2+^level by calcium assay

A colorimetric calcium detection kit (Abcam) was used to measure the Ca^2+^ level in the nigrostriatal area of the midbrain. The standards and samples were produced in accordance with the kit instructions. After being washed in cold PBS solution, 50 mg of nigrostriatal tissue was resuspended in 500 µL of Calcium Assay Buffer. Using a tissue grinder, tissue homogenization was done manually on ice. Supernatant was collected after homogenates were centrifuged at 5000 rpm for 5 min at 4 °C. The reaction mixture consisted of 90 µL of Chromogenic Reagent, 50 µL of sample, and 60 µL of Ca^2**+**^Assay Buffer, which was incubated in the dark for 10 min^90^.

### Western blot (WB) analysis

The nigrostriatal region of a mouse brain was homogenised and agitated for 2 h at 4 °C using lysis buffer (RIPA). To collect the supernatant, the homogenate was centrifuged at 12,000 rpm for 30 min and the Bradford test was used to determine the protein content. The polyacrylamide gels were loaded with the whole protein extract (30–50 µg). After that, PVDF membranes were employed for protein transblotting, and primary antibodies for TH (1:3300), α-synuclein (1:1000), Bax (1:1000), Bcl-2 (1:800), caspase-3 (1:1000), p-Akt (1:1000), Akt (1:1000), p-GSK-3β (1:1000), GSK-3β (1:1000), BDNF (1:1000), Trk-B (1:1500), CREB (1:1000) and β-actin (1:1000) were incubated overnight. The membranes were then incubated with the horseradish peroxidase (HRP-) conjugated secondary antibody for 2 h at room temperature after being washed with TBST and TBS. The Enhanced Chemiluminescence (ECL) technique was used to view the blots, and the relative density of each band was determined in comparison to that of β-actin. Blots were cut prior to hybridisation for different antibodies. Quantity One software (Version 4.6.3) was used to calculate relative density (Windows, Bio-Rad).

### Immunohistochemistry (IHC)

Prior to cutting the section of the brain, all of the procedures were carried out as described above. Using a cryotome, coronal brain slices of 15 μm thickness were cut. Tissue sections were washed twice in 0.01 M PBS (pH 7.4) for 2 min, then blocked for 1 h with 10% NGS in PBST and subsequently 1% BSA-PBST. After rinsing the sections in PBS, immunohistochemical staining for α-synuclein (1:500), BDNF(1:500), Trk-B(1:500), and p-GSK3 (1:500), was done according to the usual technique and kept it in 4 °C for 16-18 h. Thereafter, the secondary antibodies, TRITC-conjugated (anti-rabbit) and FITC-conjugated (anti-mouse), were diluted in 1% BSA-PBS and incubated for 2 h at room temperature with the corresponding primary-antibody treated tissue sections. PBS, 1% BSA-PBS, and PBS were used to wash sections at each stage. DABCO was used to mount the pieces on the slides^85^.

### Statistical analysis

The data was analysed with GraphPad Prism software (Version 5.01) and a one-way analysis of variance (ANOVA) with the Student–Newman–Keuls test and a Student's two-tailed t-test. The data is provided as mean ± standard error of the mean (SEM), with p values less than 0.05 considered statistically significant.

## Supplementary Information

Supplementary Information.

## Data Availability

Complete Raw data is provided in supplementary information.

## Abbreviations

PD: Parkinson’s disease
OLE: Oleuropein
NTs: Neurotrophins
BDNF: Brain derived neurotrophic factor
CREB: CAMP response element-binding protein
SNpc: Substantia Nigra Pars Compacta
TH: Tyrosine hydroxylase
ROS: Reactive oxygen species
DA: Dopaminergic neurons
SOD: Superoxide Dismutase
Ca^2+^: Calcium
