# Supplementary material for: Oleuropein confers neuroprotection against rotenone-induced model of Parkinson’s disease via BDNF/CREB/Akt pathway
Source: Sci Rep. 2023 Feb 11;13:2452. doi: 10.1038/s41598-023-29287-4 (PMC9922328; doi:10.1038/s41598-023-29287-4)
Supplement: Supplementary file 1 — Supplementary Information. [file 41598_2023_29287_MOESM1_ESM.docx]

**Supplementary file**

**
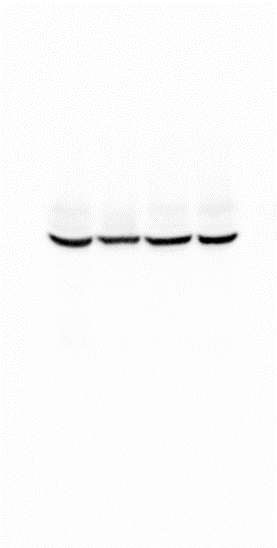
S1: TH, stripped α-synuclein and stripped β-actin**


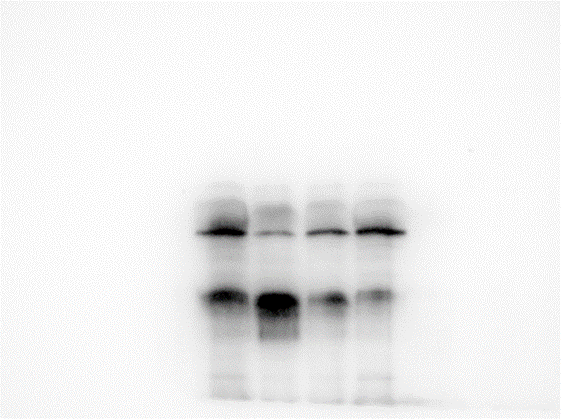
 ****
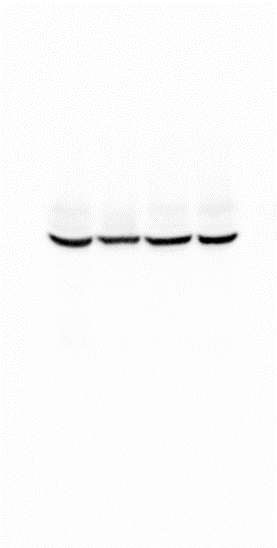


**Stripped β-actin**

**(42 kDa)**

**Stripped α-synuclein**

**(15 kDa)**

**Tyrosine Hydroxylase (TH) – 60 kDa**

**S1:** This figure shows the relative expression of α- synuclein and TH in which β-actin acts as a control. TH (8E) expression was decreased in case of rotenone- intoxicated mice. while OLE administered group showed significantly increased expression of TH. Aggregation of α-synuclein (8F) was found to be more in rotenone group than control whereas OLE-treated group showed lower aggregation of α-synuclein.

**Stripped β-actin**

**β-actin (42KDa)**

**S2: BDNF and stripped β-actin**

β-actin (42KDa)


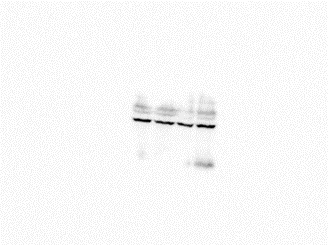


**Stripped β-actin**

**(42 kDa)**

**Brain Derived Neurotrophic Factor (BDNF) – 28 kDa**

**S2:** This figure shows the relative expression of BDNF in which β-actin acts as a control. BDNF (8B) expression was decreased in case of rotenone- intoxicated mice. while OLE administered group showed significantly increased expression of BDNF.

**S3:** **p-TrkB, TrkB and stripped β-actin**


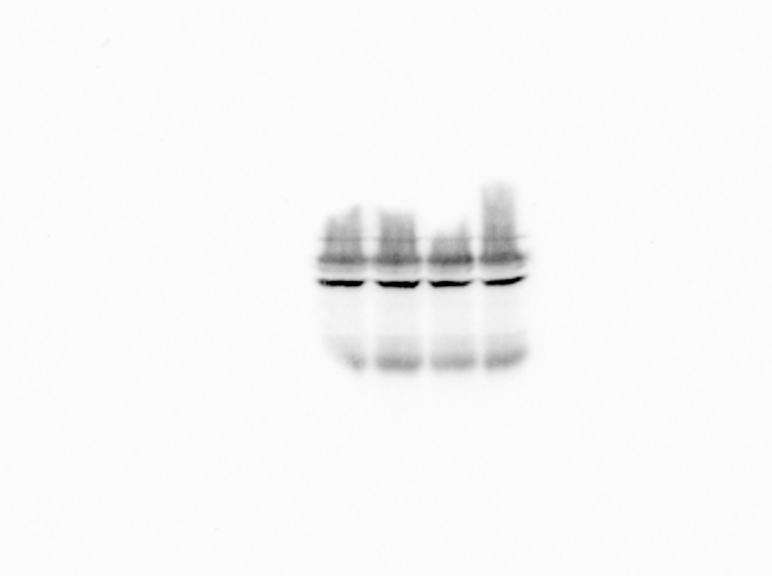


**TrkB (87 kDa)**

**Stripped β-actin**

**(42 kDa)**

**S3:** This figure shows the relative expression of Trk B in which β-actin acts as a control. Trk B (8C) expression was decreased in case of rotenone- intoxicated mice. while OLE administered group showed significantly increased expression of Trk B.

**p-TrkB (90 kDa)**


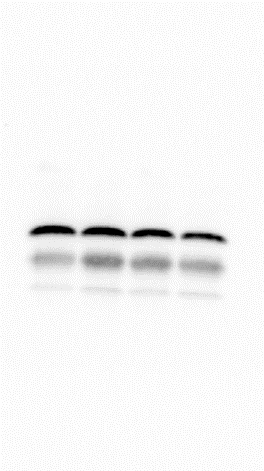
**S4: p-CREB, CREB and stripped β-actin**


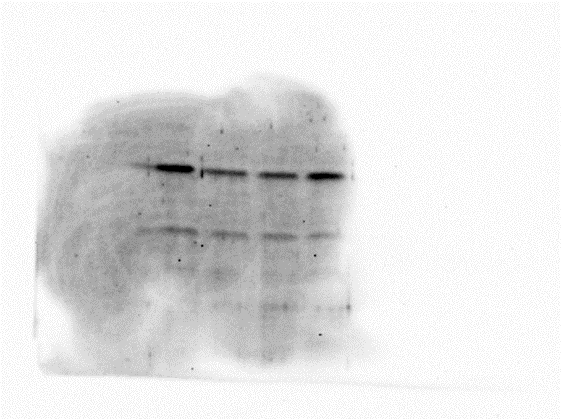


**p-CREB (43 kDa)**

**Stripped β-actin**

**(42 kDa)**

**Stripped CREB (36 kDa)**

**S4:** This figure shows the relative expression of CREB in which β-actin acts as a control. CREB (8D) expression was decreased in case of rotenone- intoxicated mice. while OLE administered group showed significantly increased expression of CREB.

**S5: p-Akt, Akt and stripped β-actin**


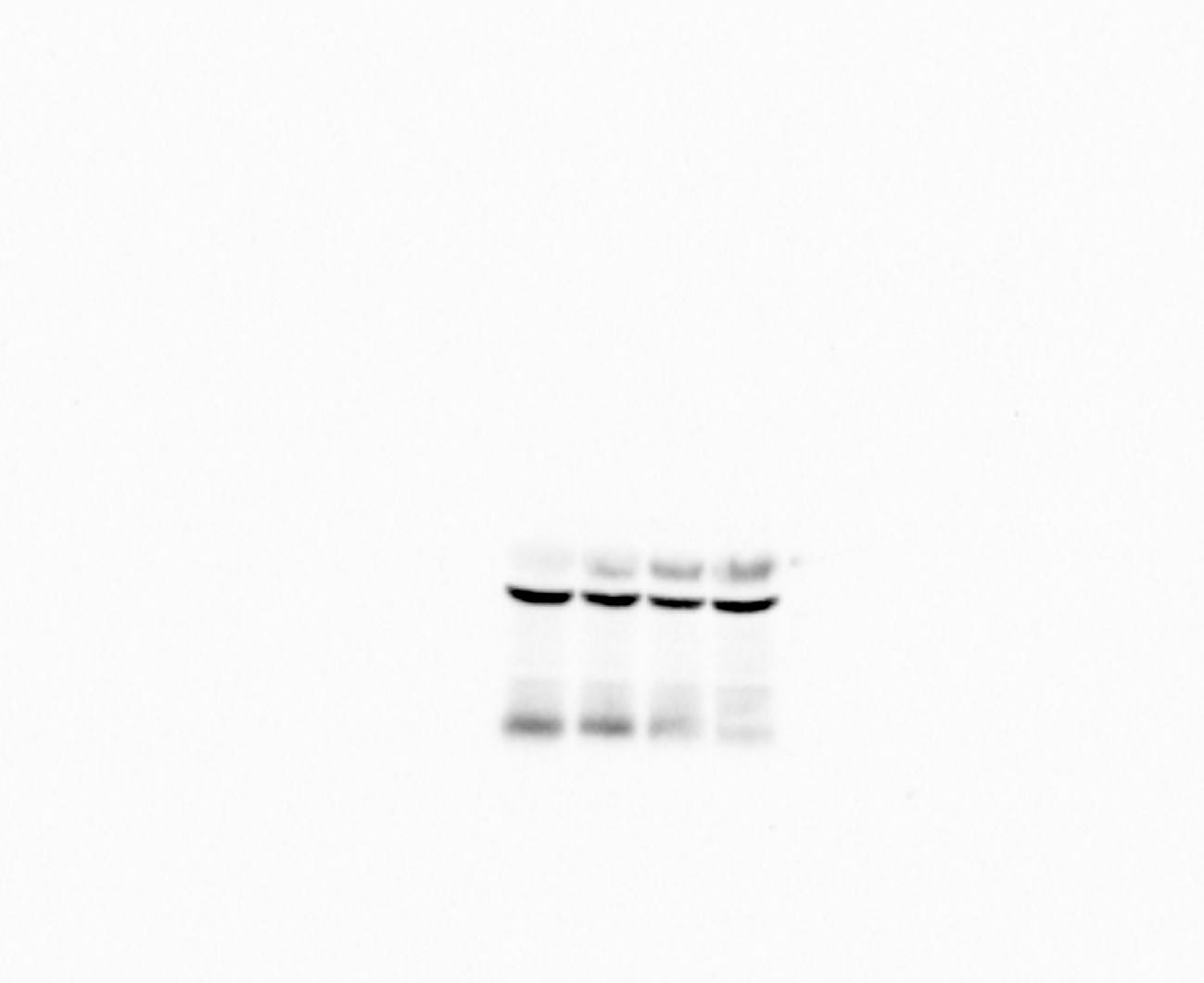


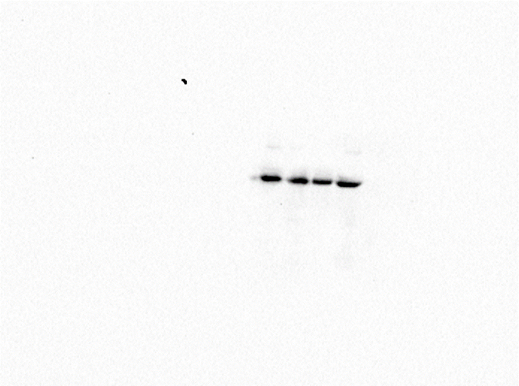


**Akt (57 kDa)**

**p-Akt (60 kDa)**

**Stripped β-actin**

**(42 kDa)**

**S5:** This figure shows the relative expression of Akt in which β-actin acts as a control. The ratio of p-Akt/Akt (9D) expression was decreased in case of rotenone-intoxicated mice. while OLE administered group showed significantly increased expression of Akt.

**S6: p-GSK-3β, GSK-3β and stripped β-actin**


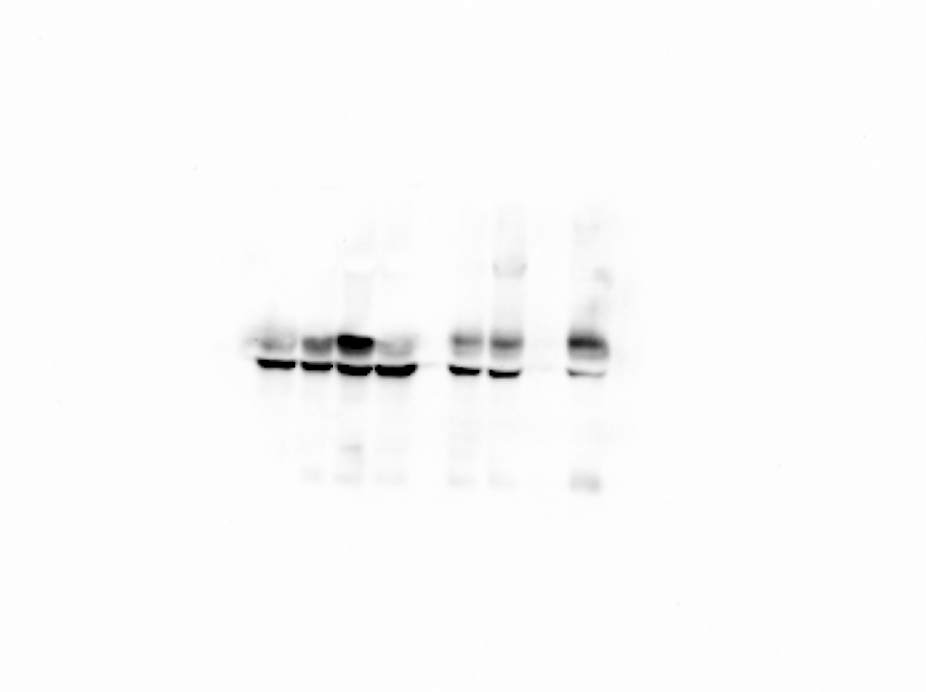





**p-GSK-3β (48 kDa)**

**Stripped GSK-3β (43 kDa)**


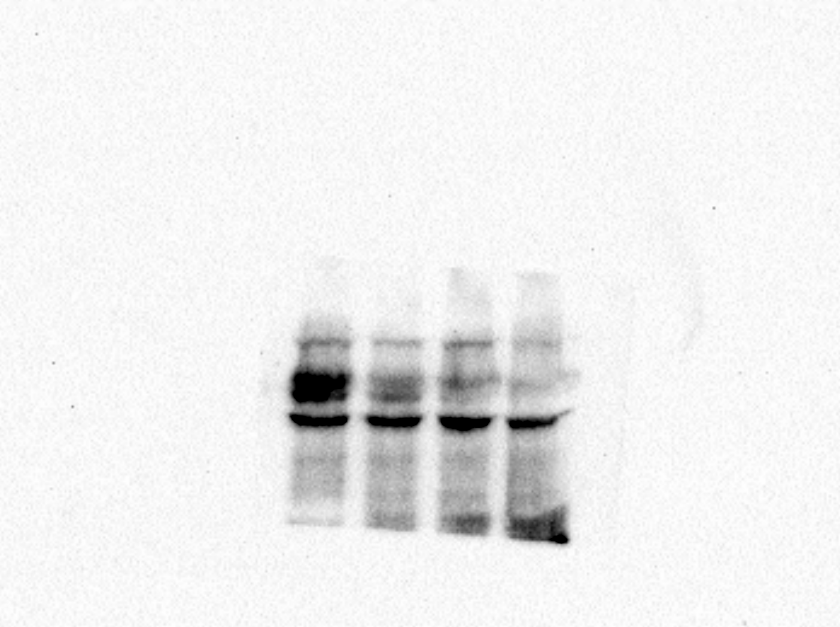


**S6**: This figure shows the relative expression of GSK-3β in which β-actin acts as a control. The ratio of p-GSK-3β/GSK-3β (9E) expression was decreased in case of rotenone-intoxicated mice. while OLE administered group showed significantly increased expression of p-GSK-3β.

**Stripped β-actin (42 kDa)**

**Stripped β-actin**

**(42 kDa)**

**S6:** This figure shows the relative expression of GSK-3β in which β-actin acts as a control. The ratio of p-GSK-3β/GSK-3β (9E) expression was decreased in case of rotenone-intoxicated mice. while OLE administered group showed significantly increased expression of p-GSK-3β.

**S7: Bax, Bcl2 and stripped β-actin**


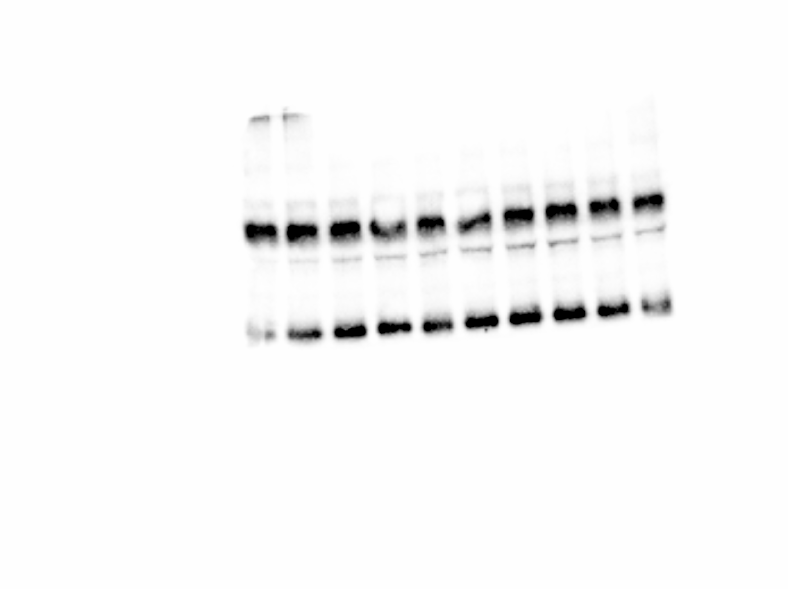

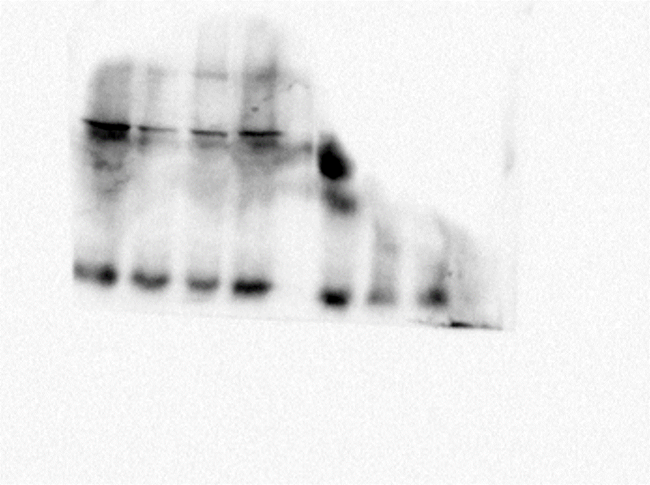

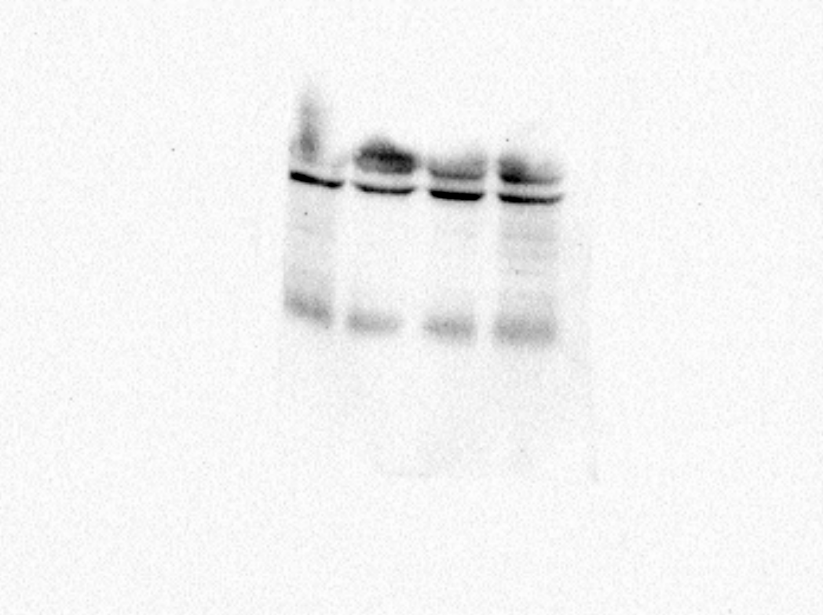

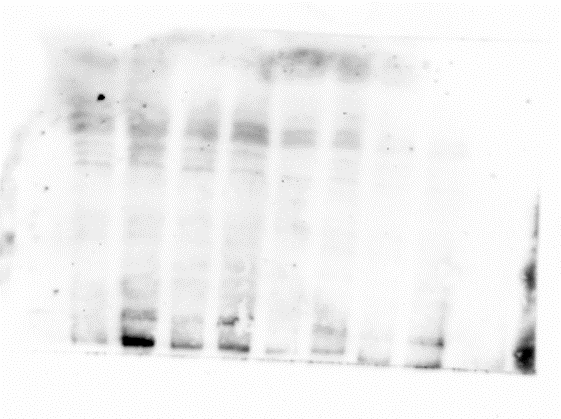


**Stripped β-actin**

**(42 kDa)**

**Bax (16 kDa)**

**Stripped β-actin**

**(42 kDa)**

**S7:** Using the Western blotting method and protein densitometry analysis, the ratio of Bax and Bcl-2 was examined in the SN of mice (9B). Rotenone induced mice showed increased in Bax/Bcl-2 ratio whereas OLE inhibited the increase in ratio of Bax/BCl_2 ._

**Bcl-2 (27 kDa)**

**S8: Cleaved caspase 3 and stripped β-actin**


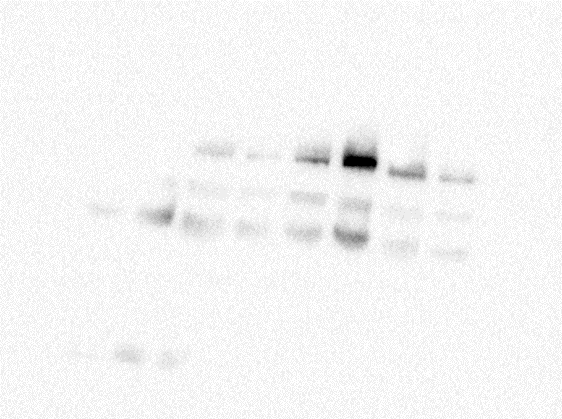

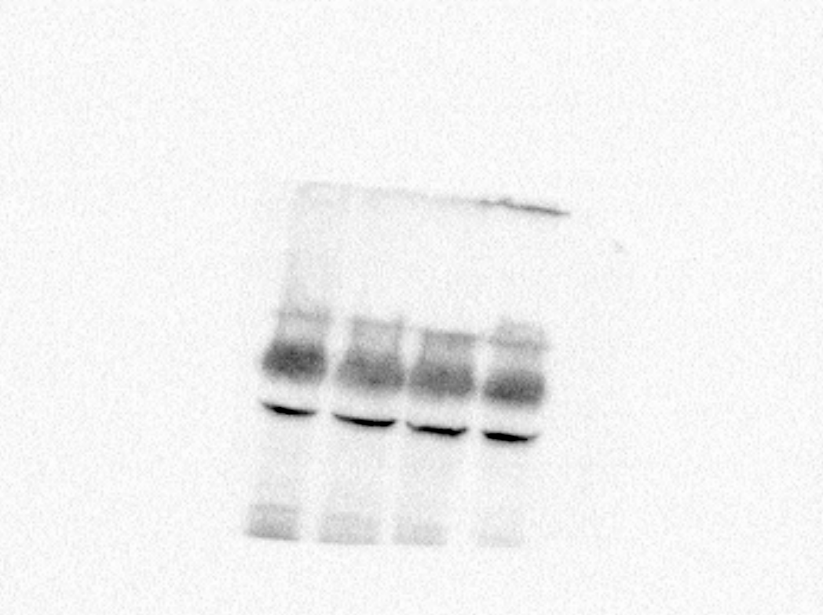


**Stripped β-actin**

**(42 kDa)**

**S8:** This figure shows the relative expression of Cleaved caspase 3 in which β-actin acts as a control. Cleaved caspase 3 (9C) expression was decreased in case of rotenone- intoxicated mice. while OLE administered group showed significantly increased expression of Cleaved caspase 3.

**Cleaved caspase 3 (35 kDa)**

**S9: Immunohistochemical staining of Tyrosine hydroxylase (TH) in SN region**


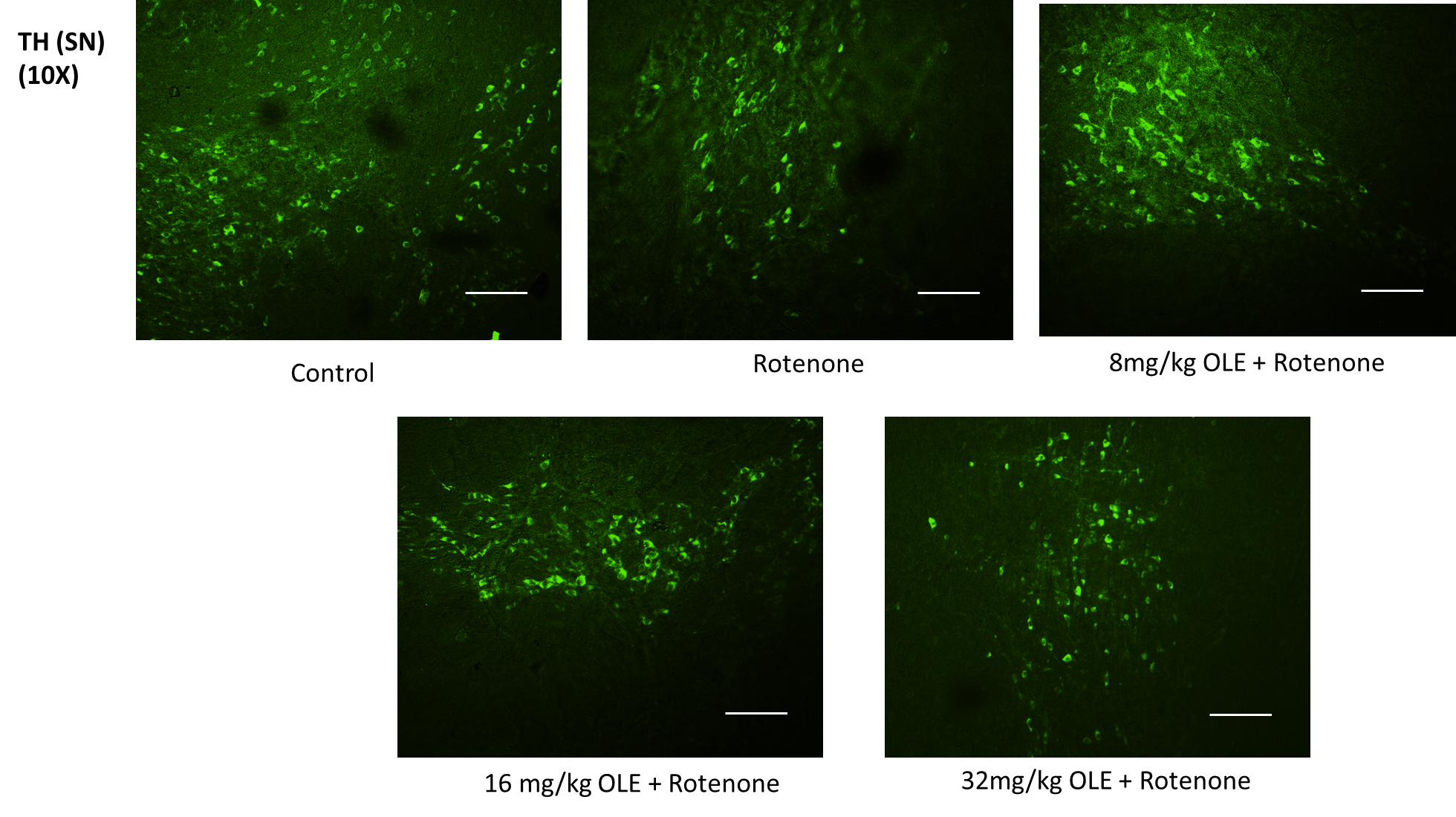


**S9:** The immunoreactivity of TH in the SN of various experimental groups was examined using immunohistochemistry under the florescence microscope (20X). Rotenone intoxicated group showed less immunoreactivity than control in SN. While, increased immunoreactivity of TH was observed in case of OLE treated group (16 and 32 mg/kg bwt) in SN. In all these three biochemical parameters, the OLE doses of 8mg/kg bwt. showed a less significant effect.

**S10: Immunohistochemical staining of Tyrosine hydroxylase (TH) in ST region**


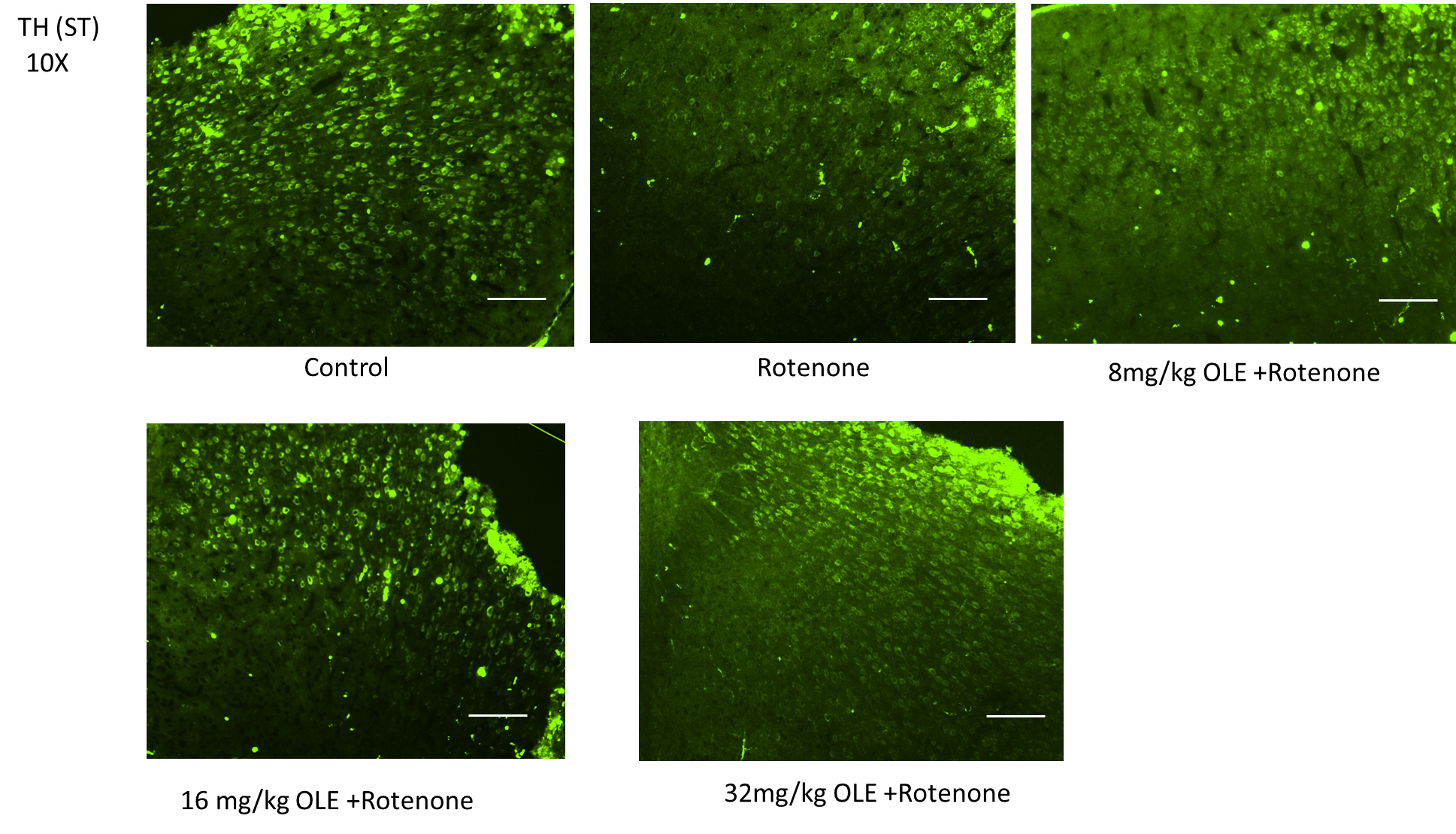


**S10:** The immunoreactivity of TH in the ST of various experimental groups was examined using immunohistochemistry under the florescence microscope (20X). Rotenone intoxicated group showed less immunoreactivity than control in ST. While, increased immunoreactivity of TH was observed in case of OLE treated group (16 and 32 mg/kg bwt) in ST. In all these three biochemical parameters, the OLE doses of 8mg/kg bwt. showed a less significant effect.

**S11: Immunohistochemical staining of Alpha-synuclein in SN region**


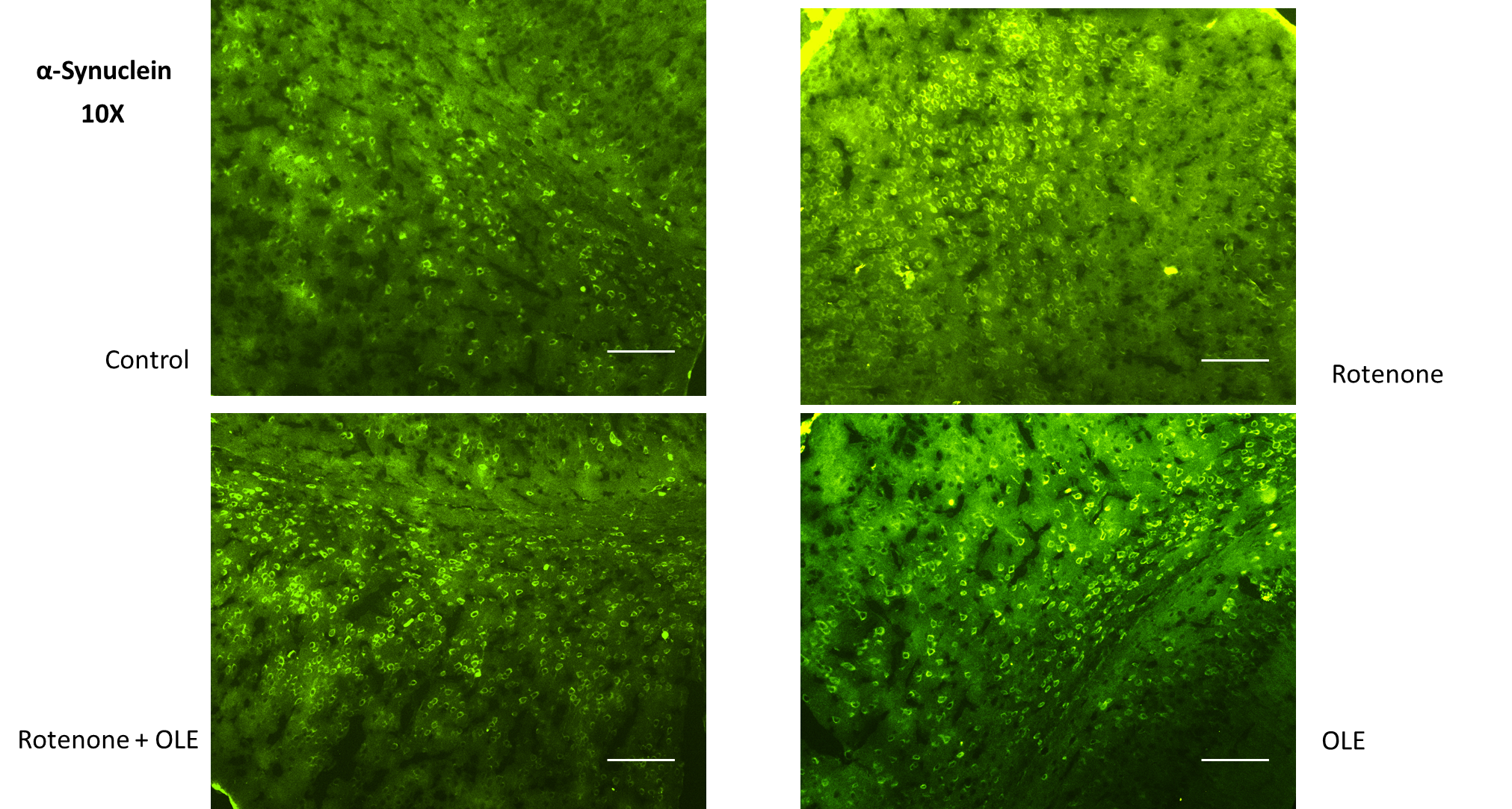


**S11:** The immunohistochemical staining of **[A]** **α- synuclein.** The increase in expression of α-synuclein in rotenone group while neuroprotection of OLE significantly decreased the expression of α-synuclein.

**S12: Immunohistochemical staining of TrkB in SN region**


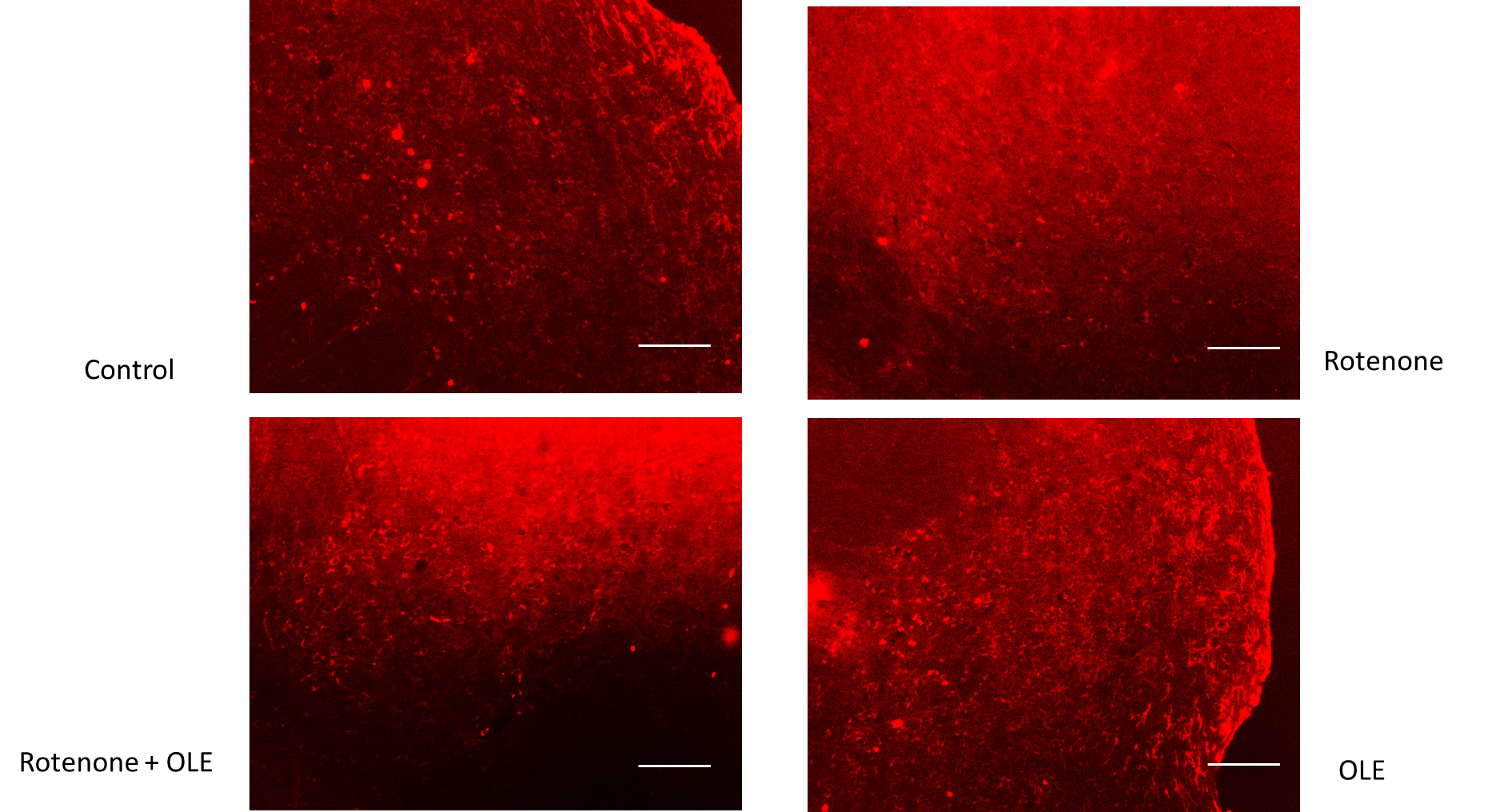


**S12:** The immunohistochemical staining of **[B]** **Trk B.** Trk B expression was reduced upon rotenone intoxication whereas, OLE significantly upregulated the expression of Trk B in OLE treated group.

**S13:** **Immunohistochemical staining of GSK-3β in SN region**

**
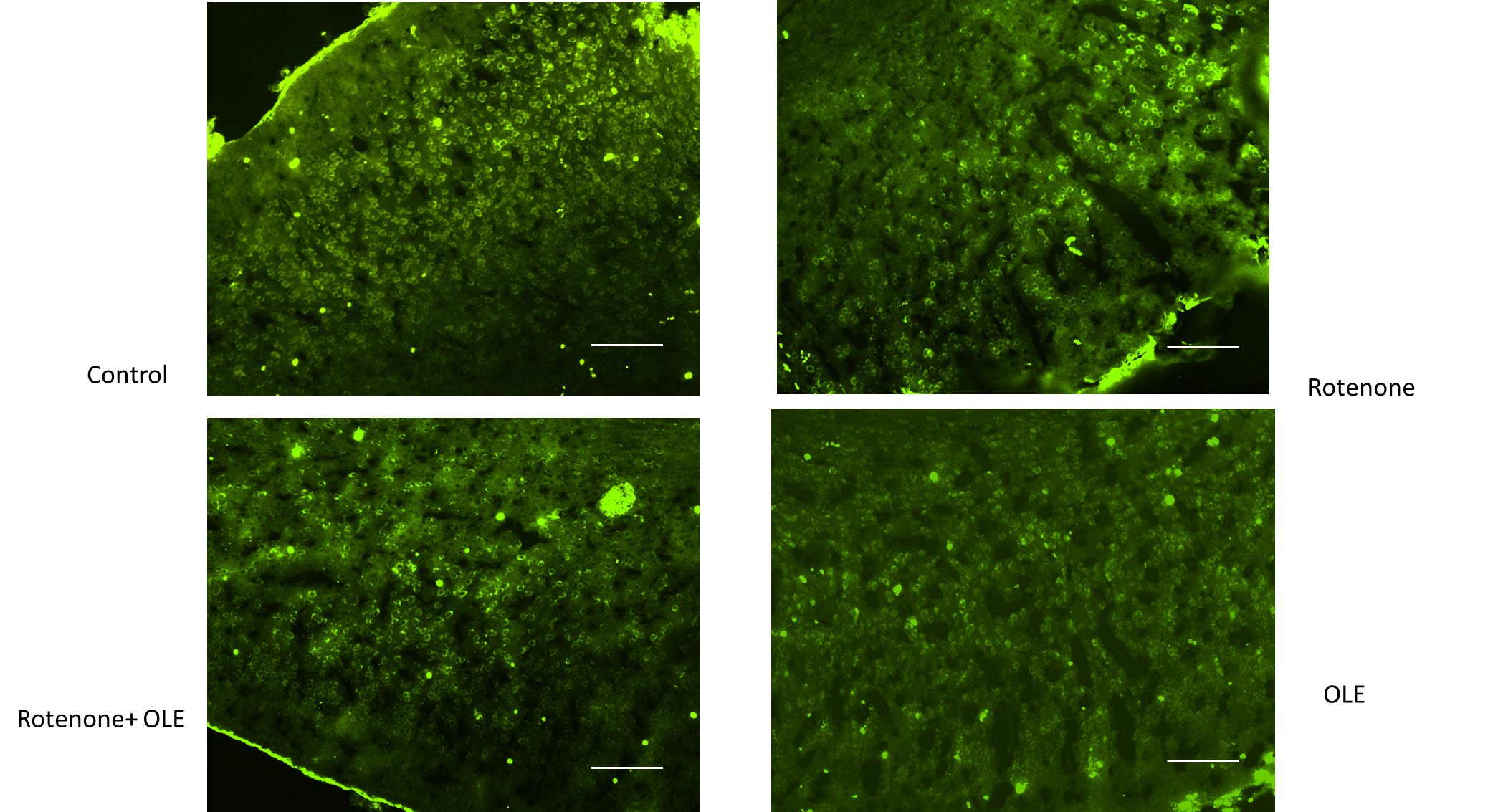
**

**S13:** In comparison with the control, immunohistochemical labelling of **[A]** p-GSK-3β. OLE treatment dramatically increased p-GSK-3β
